# Insights into the Mechanisms Underlying Ultraviolet-C Induced Resveratrol Metabolism in Grapevine (*V. amurensis* Rupr.) cv. “Tonghua-3”

**DOI:** 10.3389/fpls.2016.00503

**Published:** 2016-04-19

**Authors:** Xiangjing Yin, Stacy D. Singer, Hengbo Qiao, Yajun Liu, Chen Jiao, Hao Wang, Zhi Li, Zhangjun Fei, Yuejin Wang, Chonghui Fan, Xiping Wang

**Affiliations:** ^1^State Key Laboratory of Crop Stress Biology in Arid Areas, College of Horticulture, Northwest A&F UniversityYangling, China; ^2^Key Laboratory of Horticultural Plant Biology and Germplasm Innovation in Northwest China, Ministry of Agriculture, Northwest A&F UniversityYangling, China; ^3^Department of Agricultural, Food and Nutritional Science, University of AlbertaEdmonton, AB, Canada; ^4^College of Veterinary Medicine, Shaanxi Center for Stem Cell Engineering and Technology, Northwest A&F UniversityShaanxi, China; ^5^Boyce Thompson Institute for Plant Research, Cornell UniversityIthaca, NY, USA

**Keywords:** grape, pathways, promoter, resveratrol metabolism, transcriptome, ultraviolet-C

## Abstract

Stilbene compounds belong to a family of secondary metabolites that are derived from the phenylpropanoid pathway. Production of the stilbene phytoalexin, resveratrol, in grape (*Vitis* spp.) berries is known to be induced by ultraviolet-C radiation (UV-C), which has numerous regulatory effects on plant physiology. While previous studies have described changes in gene expression caused by UV-C light in several plant species, such information has yet to be reported for grapevine. We investigated both the resveratrol content and gene expression responses of berries from *V. amurensis* cv. Tonghua-3 following UV-C treatment, to accelerate research into resveratrol metabolism. Comparative RNA-Seq profiling of UV-C treated and untreated grape berries resulted in the identification of a large number of differentially expressed genes. Gene ontology (GO) term classification and biochemical pathway analyses suggested that UV-C treatment caused changes in various cellular processes, as well as in both hormone and secondary metabolism. The data further indicate that UV-C induced increases in resveratrol may be related to the transcriptional regulation of genes involved in the production of secondary metabolites and signaling, as well as several transcription factors. We also observed that following UV-C treatment, 22 stilbene synthase (*STS*) genes exhibited increases in their expression levels and a *VaSTS* promoter drove the expression of the *GUS* reporter gene when expressed in tobacco. We therefore propose that UV-C induction of *VaSTS* expression is an important factor in promoting resveratrol accumulation. This transcriptome data set provides new insight into the response of grape berries to UV-C treatment, and suggests candidate genes, or promoter activity of related genes, that could be used in future functional and molecular biological studies of resveratrol metabolism.

## Introduction

Stilbenes, which are a small family of secondary metabolites derived from the phenylpropanoid pathway, are produced in a taxonomically broad range of plant species and have been associated with both enhancing plant disease resistance and having a positive effect on human health as a dietary component (Chong et al., 2009). Most stilbenes are derivatives of the basic unit *trans*-resveratrol (3, 5, 4′-trihydroxy-transstilbene), although other structures have been identified in specific plant families. Resveratrol has been suggested to have health benefits associated with a moderate consumption of red wine (Siemann and Creasy, 1992), and many studies have reported that it can prevent, or slow, the progression of a wide variety of illnesses, including cancer and cardiovascular disease, and that it may even extend the lifespan of various organisms (Baur and Sinclair, 2006). As such, it is one of the most extensively studied natural products.

There is considerable evidence that supplemental UV radiation can induce the production of stilbene compounds (Berli et al., 2008; Li et al., 2008). The solar ultraviolet (UV) spectrum is continuous, but may be divided into three wavelength bands: UV-A (320–400 nm), UV-B (290–320 nm) and UV-C (200-290 nm). While UV-B radiation is potentially harmful, it is absorbed to some extent by atmospheric ozone, which reduces its ability to reach the earth's surface. UV-A, on the other hand, is not attenuated by atmospheric ozone, and this less damaging type of radiation plays an important role in plant photomorphogenesis. Short wavelength UV-C radiation is the most energetic of the three and is often described as “germicidal UV,” due to its potency against a broad range of microorganisms (Sun et al., 2006; Kasim et al., 2008). Unlike the other two forms of UV radiation, it is effectively absorbed by both oxygen and ozone in the stratosphere, such that relatively little reaches the earth's surface; however, it is being widely studied as a means to disinfect fresh fruit and vegetables to preserve their quality. As a result of the potentially damaging effects of UV radiation, plants have evolved a number of sophisticated mitigation mechanisms, such as the accumulation of UV-absorbing phenolic and flavonoid molecules in epidermal cells to reduce light penetration, and the activation of antioxidant defenses to limit photo-oxidative damage (Hollósy, 2002; Kasim et al., 2008).

Since grape (*Vitis vinifera*) was one of the first species with a high resveratrol content to have its genome fully sequenced (Jaillon et al., 2007), much current research is focused on increasing the levels of stilbenes in grape berries. Studies concerning the accumulation of resveratrol in grape, and the characterization of gene expression following UV treatment will provide important information for the breeding of crops with increased resistance to UV-C, as well as clues regarding the mechanism behind UV-induced accumulation of resveratrol. Little is known about the changes in gene expression triggered by UV-C radiation in grapevine (*Vitis* spp.), and to address this we chose *V. amurensis* Rupr. cv. Tonghua-3, which is a grapevine clone that is well adapted to the growing conditions in China, for our study. Moreover, we determined that Tonghua-3 had the highest berry resveratrol content of seven grape accessions tested, making it an ideal research material to investigate gene expression responses to UV-C radiation. HPLC was used to analyze the content of stilbene compounds in grape berries at six time points following UV-C treatment. Two of these time points (4 and 24 h) were subsequently targeted to investigate UV-C induced gene expression responses, using RNA-Seq analysis (Figure S1). Based on the data obtained, we also analyzed the responsiveness of a grape stilbene synthase (*VaSTS*) promoter to UV-C irradiation. The resulting data set provides insight into the UV-C response of grape berries, and also yields potential candidate genes that may be used for future research into resveratrol metabolism.

## Materials and methods

All chemicals were purchased from Sigma-Aldrich (St. Louis, USA) unless otherwise noted.

### Grape material

Chinese wild *V. quinquangularis* cv. “83-4-96,” “Danfeng-2” and “Shang-24,” *V. amurensis* cv. “Tonghua-3,” *V. davidii* cv. “Tangwei,” *V. vinifera* cv. “Muscat Rose,” and *V. labrusca* × *V. vinifera* cv. “Kyoho” genotypes were obtained from the Grape Repository (34°20′N, 108°24′E) of the Northwest A&F University, Yangling, Shaanxi, China. All grapevines were trained to a T-trellis with a planting density of 1.5 m between plants in 2.5 m rows. Berries were judged to be ripe based on data from the previous season's ripening dates and soluble solids levels. Approximately 300 berries total were randomly selected from each cultivar. Approximately 80–100 berries were harvested from three separate locations, respectively, with each group of 80–100 berries considered as one replicate, resulting in three total replicates in each case.

### Extraction and HPLC analysis of stilbenes/resveratrol

Stilbenes were extracted according to methods described by Sun (Sun et al., 2006) with slight modifications. In brief, 2 g of berry tissue was extracted in 10 ml 80% (v/v) methanol. The samples were then treated with ultrasound for 30 min and kept at 4°C in darkness for 12 h. The extracts were centrifuged at 6000 rpm for 15 min at 4°C, and the pellets re-extracted twice more. The three resulting supernatants were then combined and vacuum-dried using a rotary evaporator, RE-52AA (Shanghai Jinpeng Analytical Instruments Co. Ltd, Shanghai, China). The dried samples were dissolved in 1 mL of pure methanol, filtered through a 0.22 μm filter and stored at −20°C pending further analysis.

Quantitative analysis of stilbenes was performed using a Waters 600E-2487 HPLC system (Waters, USA) equipped with an Agilent ZORBAX SB-C18 column (5 μm, 4.6 × 250 mm). The column temperature was set at 25°C and detection of stilbenes was achieved at 306 nm with a flow rate of 0.8 ml/min. The solvent system consisted of 1 min in isocratic 20% aqueous acetonitrile, 30 min in a linear gradient from 20 to 75% aqueous acetonitrile, 2 min in a linear gradient from 75 to 100% acetonitrile, 3 min in isocratic 100% acetonitrile, 1 min in a linear gradient from 100 to 20% aqueous acetonitrile, and 10 min in isocratic 20% aqueous acetonitrile. *Trans*-resveratrol was purchased from Sigma-Aldrich while *cis*-resveratrol was obtained from *trans*-resveratrol by conversion using UV-C irradiation (Wang et al., 2013b).

### UV-C irradiation

Mature grape berries were irradiated with UV-C (254 nm, Spectroline, Model ZQJ-254, output 300 mW/cm^2^) after harvest for 10 min at a distance of 15 cm. Negative controls consisted of non-irradiated material. Following irradiation, both UV-treated and control plants were transferred to a Robert manual incubator (Model PRX-350D), and incubated in the dark at 25°C with a relative humidity of 80% for 60 h. After each incubation period (0, 4, 12, 24, 36, 48, and 60 h) a subset of berries was collected, frozen in liquid nitrogen and stored at −80°C. Each treatment was performed with at least three independent replicates, and each replicate consisted of randomized plant material.

### RNA extraction

Two biological replicates of each treatment were used for all RNA-Seq experiments. Total RNA was extracted from whole berries of Chinese wild *V. amurensis* cv. Tonghua-3 using an SDS/phenol method (Zhang et al., 2003). RNA quality and quantity were monitored on a 1.2% denatured agarose gel and with a NanoDrop 1000 Spectrophotometer (Thermo Scientific, Wilmington, DE, USA), respectively. Treated berries were defined as those that had been irradiated with UV-C and frozen in liquid nitrogen 4 or 24 h post-treatment, while non-irradiated berries at the same time points were used as controls.

### RNA-seq data analysis

Approximately 10 μg cDNA from each of the eight samples (two biological replicates for each treatment) were sequenced in the Genomics Facility at Cornell University's Biotechnology Resource Center (http://www.biotech.cornell.edu/brc/genomics-facility). Strand-specific RNA-Seq libraries were constructed as previously described (Zhong et al., 2011) and sequenced on an Illumina HiSeq 2000 system in the single-end mode. The length of the reads was 100 bp. RNA-Seq reads were first aligned to ribosomal RNA sequences using Bowtie (Langmead et al., 2009) and the aligned reads were removed. The resulting filtered reads were then aligned to the grape genome sequence using TopHat (Trapnell et al., 2009). Following alignment, the count of mapped reads from each sample was derived and normalized to RPKM (reads per kilobase of exon model per million mapped reads). Differentially expressed genes (DEGs) between UV-C treated and control samples at each time point were identified using the DESeq 1.8.3 package (Anders and Huber, 2010) with the raw count data. Raw *P*-values were adjusted for multiple testing using a false discovery rate (FDR; Benjamini and Hochberg, 1995). Genes with an FDR value of < 0.05 and a fold-changes > 2.0 were considered to be DEGs. Correlations between the 4 h and 24 h time points, along with the untreated control samples, were investigated using cluster analysis with the Cluster 3.0 software.

### Gene ontology (GO) term analysis and pathway prediction

Multi-array log2 transformation, normalization, and *t*-tests were performed to identify DEGs between any two selected groups using the geWorkbench genomic analysis software suite (http://www.geworkbench.org) (Floratos et al., 2010). Hierarchical clustering was performed using Pearson's metrics and total linkage algorithms. Gene Ontology and Pathway analysis of co-expressed genes was performed using Plant MetGenMAP (http://bioinfo.bti.cornell.edu/cgi-bin/MetGenMAP/home.cgi) (Joung et al., 2009). To adjust the *P*-value, a hypergeometric test with the Benjamini and Hochberg false discovery rate (FDR) was performed using the default parameters.

### Validation of RNA-seq data by quantitative real-time RT-PCR (qRT-PCR)

First-strand cDNA was synthesized from 1 mg DNase-treated (TaKaRa Biotechnology) total RNA with the PrimeScript™ II 1st Strand cDNA Synthesis Kit (TaKaRa Biotechnology, Dalian, China). Quantitative real-time RT-PCR was conducted using SYBR green (TaKaRa Biotechnology) on an IQ5 real-time PCR machine (Bio-Rad, CA, USA). Each reaction was carried out in triplicate with a reaction volume of 25 μl. Cycling parameters were 95°C for 30 s, 40 cycles of 95°C for 5 s, and 60 °C for 30 s. For dissociation curve analysis, a program including 95°C for 15 s, followed by a constant increase from 60°C to 95°C, was included after the PCR cycles. The expression of grape *ACTIN1* (GenBank Accession number AY680701), amplified with primers F (5′ - GAT TCT GGT GAT GGT GTG AGT - 3′) and R (5′ - GAC AAT TTC CCG TTC AGC AGT - 3′), as well as grape *GAPDH* (GenBank Accession number CB973647), amplified with primers F (5′ - TTC TCG TTG AGG GCT ATT CCA - 3′) and R (5′ - CCA CAG ACT TCA TCG GTG ACA - 3′), were used as internal controls. Relative expression levels were analyzed using the IQ5 software and the normalized-expression method.

### Cloning of the *VaSTS* gene promoter

A stilbene synthase (*VaSTS*) promoter region was isolated by PCR using Chinese wild *V. amurensis* genomic DNA as a template. Genomic DNA was extracted with phenol/chloroform, precipitated with ethanol and then stored at −40°C for subsequent cloning. Primary PCR with the gene-specific F (5′ - GAA TTC AAT AAA TCT AAT AAA TAT T - 3′) and R (5′- GAC GTC AAT CAG ACT GGT AGA TAC AG - 3′) primers was performed to amplify the randomly selected *VaSTS* gene (GSVIVT01010565001) promoter, which was then ligated into the cloning vector pMD 19-T (TaKaRa Biotechnology, Dalian, China) and transformed into *Escherichia coli* strain DH5a (TaKaRa Biotechnology, Dalian, China). A positive candidate clone was sequenced at Invitrogen Biotechnology (Invitrogen Biotechnology, Shanghai, China), and the promoter sequence was analyzed using the PlantCARE (http://bioinformatics.psb.ugent.be/webtools/plantcare/html/) database (Lescot et al., 2002).

### Construction of *VaSTS*::*GUS* fusion vectors

Two expression vectors, pC0380GUS and pC35SGUS (Xu et al., 2010), were used for transient expression assays. The 1,570bp upstream region of *VaSTS* was amplified by PCR as described above, with *Pst*I and *Eco*RI restriction enzyme sites introduced at the 5′ ends of each primer, respectively. The *VaSTS* promoter fragment was then double-digested with *Pst*I/*EcoR*I, and ligated into the corresponding site of the pC0380GUS vector, which had been double-digested with the same restriction enzymes. The fusion construct was verified by sequencing and introduced into *Agrobacterium tumefaciens* strain GV3101 using the freeze-thaw method.

### *Agrobacterium*-mediated transient expression assays and UV-C irradiation of tobacco plants

*Agrobacterium*-mediated transient expression assays were performed as previously described (Sparkes et al., 2006) utilizing the promoter-*GUS* fusion constructs. Fully expanded, infiltrated tobacco leaves were collected from intact tobacco (*Nicotiana tabacum*) plants harboring each construct and subsequently used to evaluate GUS activity via both histochemical and qRT-PCR analyses. Forty-eight hours following *A. tumefaciens* infiltration, tobacco leaves were treated with UV-C by irradiating (254 nm, Spectroline, Model ZQJ-254, output 300 mW/cm^2^) for 15 min at a distance of 15 cm, while controls consisted of non-irradiated material. Leaves were sampled at the indicated times for qRT-PCR analysis, or 24 h after *A. tumefaciens* inoculation for GUS assays.

### qRT-PCR analysis of *GUS* transcript levels

*GUS* transcript levels in UV-C treated or untreated tobacco leaves were quantified by two-step qRT-PCR. Total RNA was isolated from tobacco leaf samples using the E.Z.N.A. ® Plant RNA Kit (Omega Bio-tek, USA, R6827-01), and the RNA concentration was adjusted to 500 ng for reverse transcription. The reactions were carried out in triplicate in a final volume of 25 μl using an iCycler iQ5 thermal cycler (Bio-Rad, Hercules, CA, USA), and a SYBR Premix Ex^*TM*^ TaqII kit (Takara). Cycling parameters were 95°C for 30 s, followed by 40 cycles of 95°C for 5 s and 62°C for 30 s. The tobacco ubiquitin gene (*UBI*, GenBank Accession number U66264) was used as the internal control. *GUS* transcript levels were calculated using the normalized-expression method. The following primers were used for real-time PCR amplifications: 5′ - ATTATGCGGGCAA CGTCTGGTATCAG - 3′ and 5′ - CAT CGGCTTCA AATGGCGTAGC - 3′ for *GUS*, 5′ - ATGAACGC TGGCGGCATG CTTA - 3′ and 5′ - AGATCTGCATTC CTCCCCTCGCTA - 3′ for *UBI*.

### GUS activity assays

Histochemical and quantitative GUS assays were carried out according to the procedure published by Jefferson (1987). Histochemical staining of the leaves was performed as previously described (Xu et al., 2010). Quantitative GUS assays, using 4-methyl umbelliferyl glucuronide (MUG; Sigma-Aldrich) as a substrate, were quantified using a Hitachi 850 fluorescence spectrophotometer (Hitachi, Tokyo, Japan), and protein concentrations were measured using the protein–dye binding assay (Bradford, 1976) and a Nicolet Evolution 300 UV-VIS spectrophotometer (Thermo Electron Corp., Madison, WI, USA) with bovine serum albumin as the standard. GUS activity was expressed as nM of 4-methylumbelliferone (4-MU; Sigma-Aldrich) generated per minute per milligram of soluble protein. Data were subjected to one-way ANOVA for comparison of the means, and one-sided paired *t*-test were performed to assess significant differences between mock and treatment conditions using the SigmaPlot 10.0 statistical software package (Ashburn, VA, USA). Differences at *P* < 0.05 were considered significant.

## Results

### Genotypic differences in *trans*-resveratrol content in grape berries

Berries from a total of 7 grape accessions, corresponding to five wild species, were analyzed for resveratrol content (Table 1). The highest levels of resveratrol were found in *V. amurensis* Tonghua-3 (2.19 μg/g FW at maturity); therefore, this genotype was chosen for further study.

**Table 1:** **Resveratrol concentrations in grape berries of different species and cultivars at different developmental stages**.

**Species**	**Accessions or cultivars**	**Content of *trans*-res (μg/g FW)**		
		**Green**	**Veraison**	**Mature**
*Vitis quinquangularis*	83-4-96	–	0.60 ± 0.08	1.57 ± 0.02
	Danfeng-2^a^	–	0.95 ± 0.09	1.16 ± 0.03
	Shang-24	0.28 ± 0.06	0.32 ± 0.05	1.94 ± 0.03
*V. amurensis* Rupr.	Tonghua-3	–	0.50 ± 0.06	2.19 ± 0.06
*V. davidii* Foex.	Tangwei	–	–	–
*V. vinifera* L.	Muscat Rose	–	0.28 ± 0.09	1.14 ± 0.02
*V. vinifera × V. labrusca*	Kyoho	–	0.69 ± 0.04	1.09 ± 0.04

a *Berry skin color of all accessions was black except for Danfeng-2 which had green skin. –Indicates not detected in sample*.

### Changes in *trans*- and *cis*-resveratrol content in response to UV-C irradiation of grape berries

**Figure 1:**
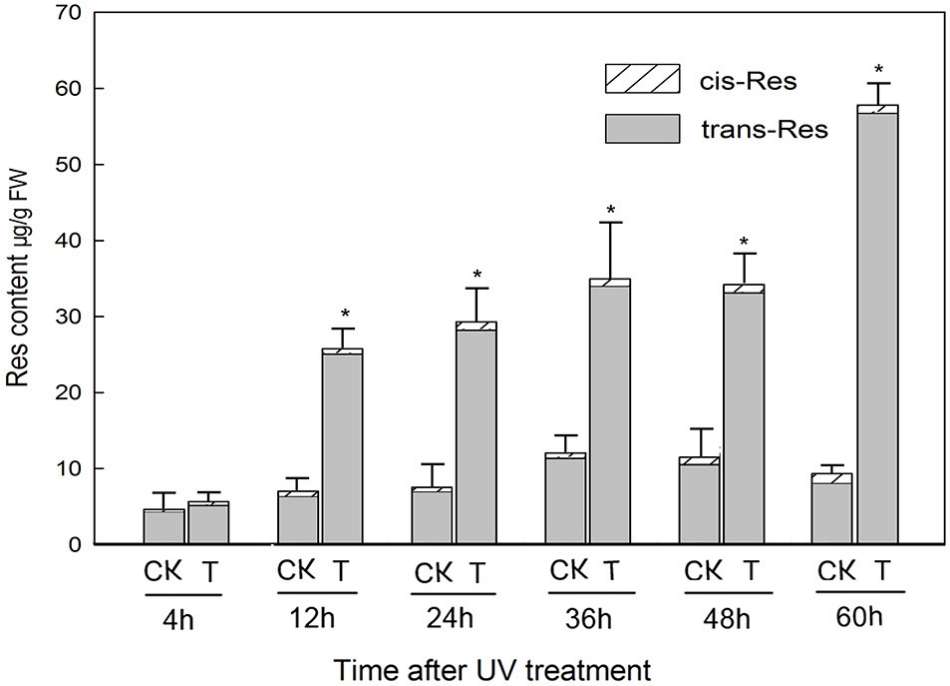
**Resveratrol (res) content of grapevine *Vitis amurensis* cv. Tonghua-3 berries at various time points after UV-C irradiation**. Each block denotes the mean value for each time point and treatment (*n* = 3), bars represent the standard error. Asterisks indicate significant differences between UV-C treated (T) and untreated control (CK) samples from the same time point (^*^P < 0.05, Paired *t*-test).

Following irradiation treatment of mature berries, *trans*-resveratrol levels were significantly higher by 12 h post-treatment than non-treated samples and continued to increase steadily until 60 h post-treatment, when the concentration reached 56.76 μg/g FW (Figure 1). Conversely, the content of *cis*-resveratrol at each time point measured did not change significantly in response to the UV-C treatment (Figure 1).

### RNA-seq analysis of the grape berry transcriptome following UV-C treatment

The transcriptome of mature grape berries was analyzed in response to UV-C treatment 4 and 24 h post-irradiation. At these two time points, *trans*-resveratrol levels were approximately equal to, and 4-fold greater than, those of the untreated control, respectively. Low quality, adaptor and barcode sequences, as well as possible rRNA reads were removed to obtain clean reads (Table S1). The percentage of reads mapping to the grape genome was approximately 70%, with the exception of repeat 1 of the untreated 4 h sample (4h-CK1) (60.12%) and repeat 2 of the UV-C treated 4 h sample (4h-T2) (50.34%). As confirmation of the robustness of the RNA-Seq dataset, we found that the two biological replicates were in good agreement with respect to gene expression levels, with 0.95 < *R*^2^ < 0.99 (Table S2).

**Figure 2:**
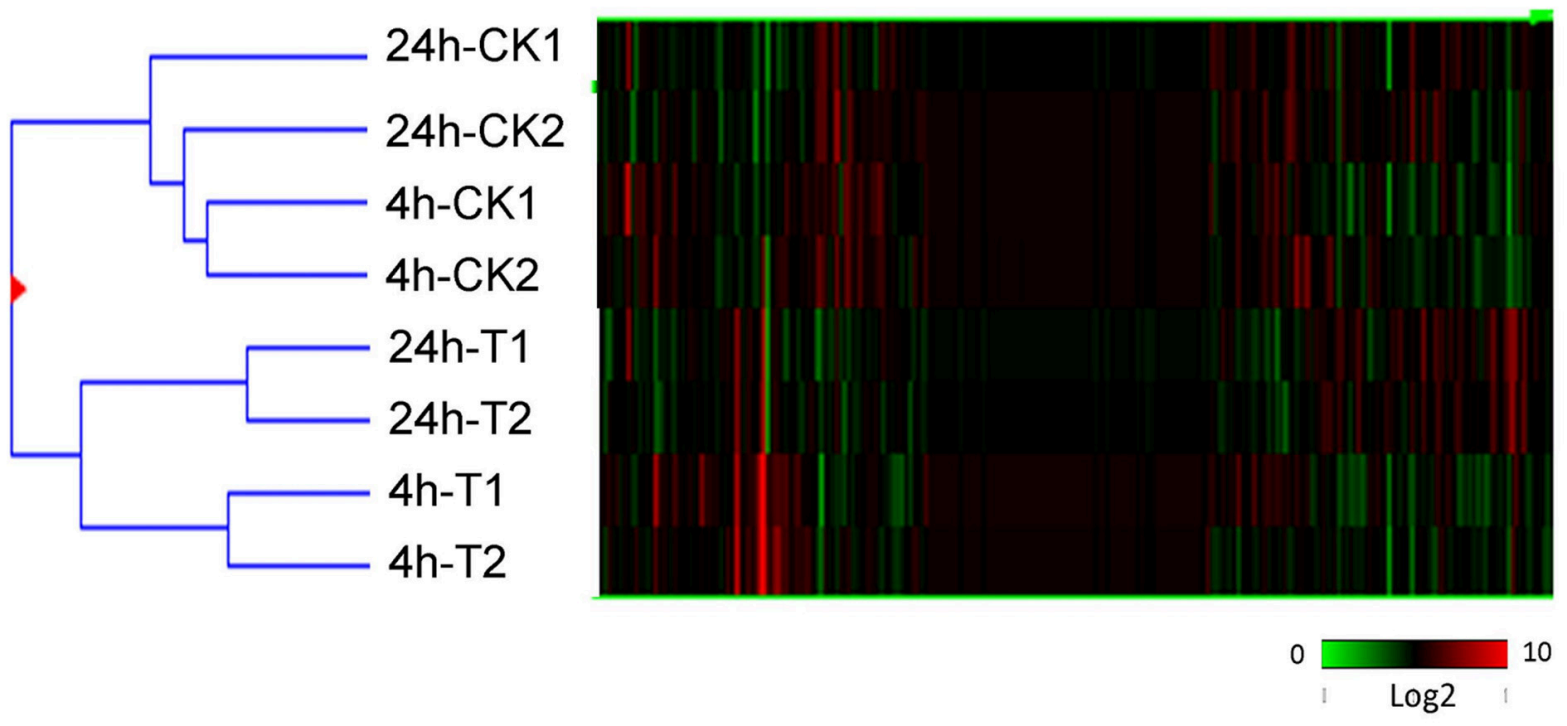
**Hierarchical cluster analysis of all transcripts identified through RNA-Seq analysis of UV-C treated (T) and untreated control (CK) berries 4 and 24 h following irradiation**. Two biological replicates (1 and 2) were used in each case. Different shades of red and green express the extent of the change according to the color bar provided (log2 ratio of control); black indicates no change.

Correlations between the 4 and 24 h post-treatment time points, along with the untreated control samples, were investigated using cluster analysis (Figure 2; Eisen et al., 1998). As expected, the 4h-CK and 24 h-CK samples (untreated controls) exhibited fewer differences in gene expression between them than did 4h-T and 24h-T samples (UV-C irradiated) (Figure 2). Interestingly, there were even more substantial differences between the two UV treated samples and their untreated controls, respectively, which correlated well with our resveratrol content results.

### Identification of DEGs by RNA-seq

Gene expression levels were measured as RPKM values and genes exhibiting an FDR < 0.05 and a fold-change in transcript expression levels > 2.0 between UV-C treated and untreated controls were regarded as DEGs. We identified 785 genes with increased transcript abundance and 97 genes with decreased transcript abundance in grape berries 4 h after UV-C treatment compared to untreated controls, with an even larger number of genes (819 with increased transcript abundance and 239 with decreased transcript abundance) 24 h after UV-C treatment. Of the 785 genes with increased transcript abundance at 4 h, 401 exhibited differences in expression at 24 h post-treatment, whereas only 54 down-regulated genes displayed differences in expression between 4 and 24 h post-treatment (Figure S2, Tables S3, S4).

### Go functional classification of the grape berry transcriptome following UV-C treatment

**Figure 3:**
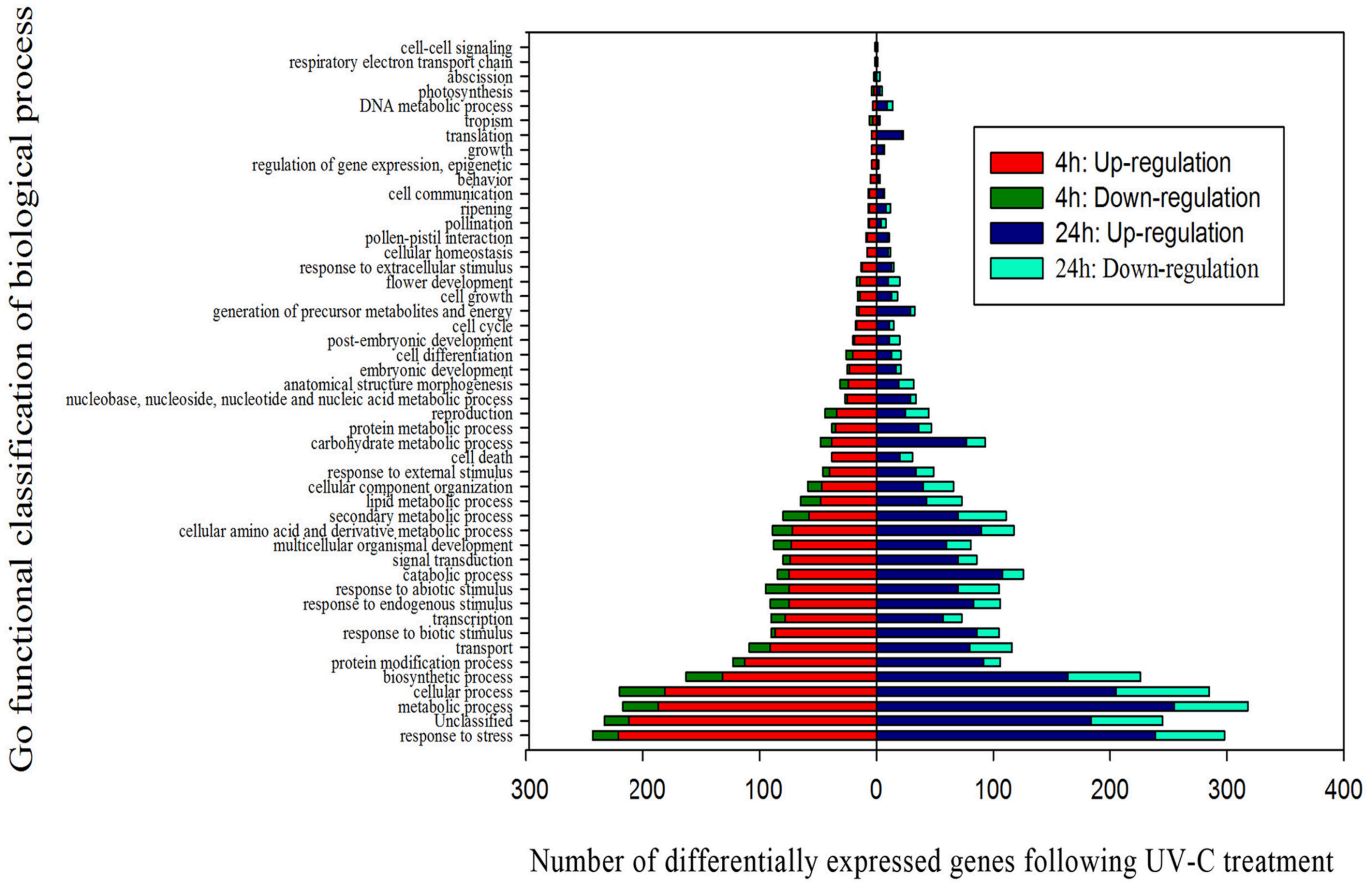
**Functional categorization of genes differentially expressed after UV-C treatment based on the “biological process” category of Gene Ontology (GO)**.

A total of 662 of the 881 DEGs identified at 4 h post-treatment, and 824 of the 1058 DEGs identified at 24 h post-treatment, were assigned to at least one term within the GO categories of “molecular function,” “cellular component,” or “biological process.” These DEGs were further classified into 100 functional subcategories (Figure 3; Table S5).

Of the genes showing increased expression 4 h post-irradiation in the “biological process” category, “response to stress” and “metabolic processes” were the most represented terms, indicating that extensive metabolic activity was taking place in the grape berries following UV-C treatment. Within the “molecular function category,” the “catalytic activity,” “protein binding” and “binding” terms were prominently represented, while the “membrane” and “plasma membrane” terms dominated the “cellular component” category (Figures S3, S4; Tables S6, S7).

We further identified GO terms within the “biological process” category that were over-represented (*p* < 0.05) both 4 and 24 h after UV-C treatment (Tables 2, 3). These GO terms served as indicators of significantly different biological processes occurring within berries following UV-C treatment, compared to their untreated control counterparts. GO terms such as “metabolic processes,” “cellular processes,” “cellular metabolic processes” and “biosynthetic processes” were enriched in the annotations of both sets of transcripts from the two time points, suggesting that a set of longer term biological activities are required to repair UV-C damage. However, we also noted a few striking differences between enriched GO terms from the two time points. For example, GO terms related to “protein binding,” “nucleotide binding,” “purine nucleotide binding” and “adenyl nucleotide binding” were highly enriched in DEGs 4 h post-treatment, but not at 24 h post-treatment (Tables 2, 3).

**Table 2:** **Significantly represented GO terms in the “biological process” category in the pool of differentially expressed genes 4 h following UV-C treatment**.

**Gene ontology term**	**Observed^a^ (%)**	**Expected^b^ (%)**	**Raw *P*-value**	**Corrected *P*-value**
Metabolic process	57.8	43.9	2.46E-17	0
Cellular process	57.3	47.3	8.31E-10	0
Cellular metabolic process	48.1	37.6	7.12E-11	0
Primary metabolic process	46.5	36.7	6.54E-10	0
Response to stimulus	34.8	20.5	6.05E-24	0
Response to stress	27.4	14.4	1.31E-24	0
Biosynthetic process	26.9	18.5	2.70E-10	0
Cellular biosynthetic process	23.5	17.5	2.34E-06	0
Response to chemical stimulus	18.8	9.9	1.99E-16	0
Developmental process	15.2	11.8	0.00101	0.00817
Defense response	15.1	6.2	1.26E-21	0
Biopolymer modification	14.1	9.4	3.02E-06	0
Protein modification process	14.0	8.9	3.17E-07	0
Post-translational protein modification	13.2	8.2	2.08E-07	0
Phosphate metabolic process	11.9	6.9	2.91E-08	0

a *The frequency of occurrence of a GO term in the tested gene list*.

b *The frequency of occurrence of a GO term in the list of genes for which probes were present on the genome*.

**Table 3:** **Significantly represented GO terms in the biological process category in the pool of differentially expressed genes 24 h following UV-C treatment**.

**Gene ontology term**	**Observed^a^ (%)**	**Expected^b^ (%)**	**Raw *P*-value**	**Corrected *P*-value**
Metabolic process	63.1	43.9	1.34E-37	0
Cellular process	57.1	47.3	4.50E-11	0
Cellular metabolic process	49.2	37.6	3.15E-15	0
Primary metabolic process	46.0	36.7	1.26E-10	0
Response to stimulus	33.9	20.5	2.62E-25	0
Biosynthetic process	28.7	18.5	1.02E-16	0
Response to stress	28.0	14.4	4.40E-32	0
Cellular biosynthetic process	24.1	17.5	2.02E-08	0
Response to chemical stimulus	17.6	9.9	2.98E-15	0
Catabolic process	11.9	6.6	4.86E-11	0
Carboxylic acid metabolic process	11.2	4.9	1.77E-17	0
Organic acid metabolic process	11.2	4.9	1.99E-17	0
Cellular catabolic process	11.2	5.8	4.86E-12	0
Cellular amino acid and derivative metabolic process	11.1	4.7	2.14E-18	0
Defense response	4.4	6.2	1.68E-22	0

a *The frequency of occurrence of a GO term in the tested gene list*.

b *The frequency of occurrence of a GO term in the list of genes for which probes were present on the genome*.

### UV-C induced pathways in grape berries

To better understand the functional roles of UV-C responsive genes involved in different metabolic pathways, we identified biochemical pathways affected by UV-C irradiation based on our expression profiling analysis. A total of 51 and 76 biochemical pathways were significantly influenced by UV-C treatment (*p*-value < 0.05) at 4 and 24 h post-treatment, respectively, (Figure 6 and Table S8). These pathways encompassed the biosynthesis or degradation of diverse metabolites including hormones, sugars and polysaccharides, amino acids, fatty acids and lipids, and secondary metabolites.

**Figure 4:**
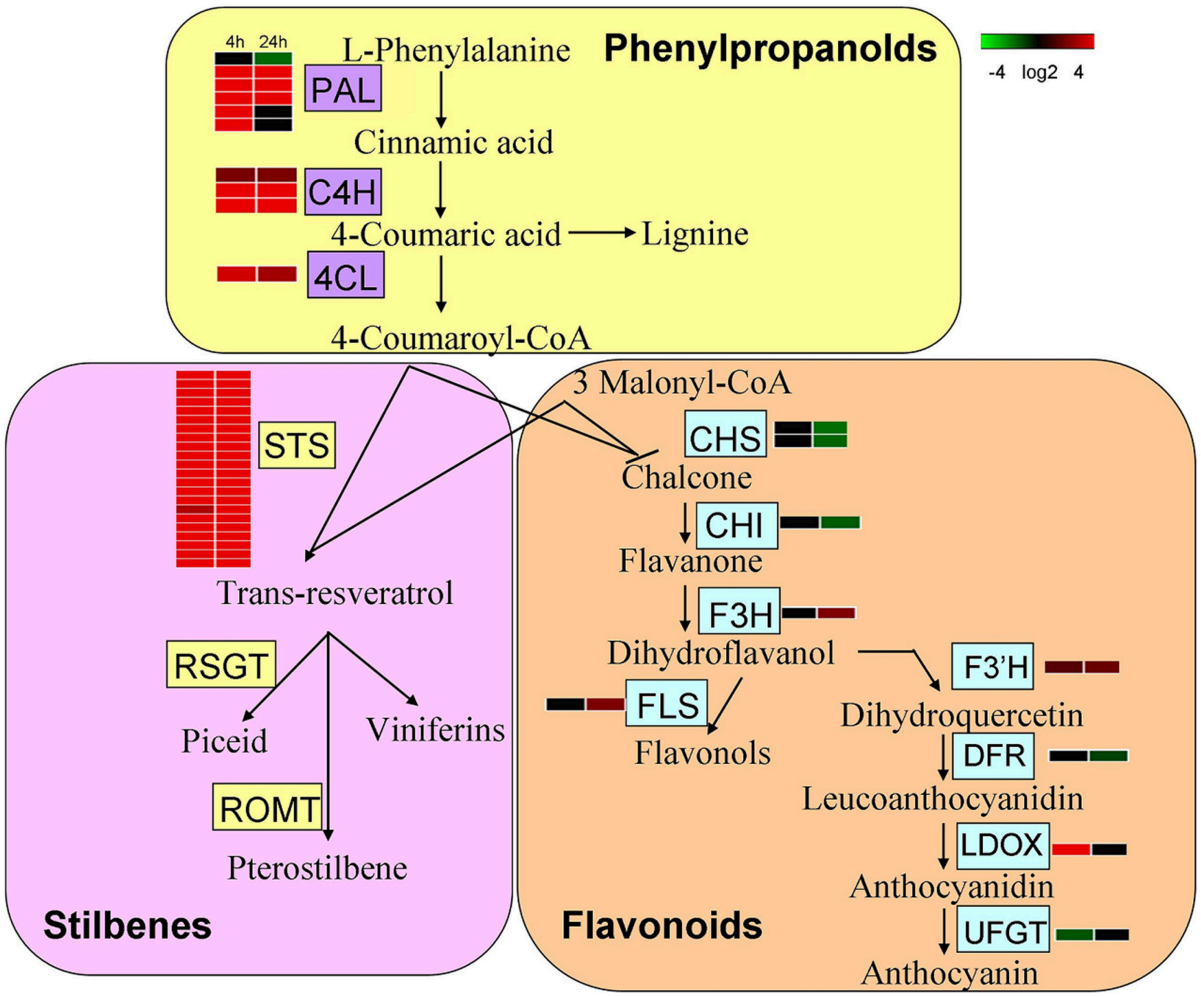
**Expression of genes involved in secondary metabolism after UV-C irradiation**. The schematic representation was determined by transcriptome data integration. Different shades of red and green express the extent of change according to the color bar provided (log2 ratio of control); black indicates no change. PAL, Phenylalanine ammonia-lyase; 4CL, 4-coumarate:CoA ligase; C4H, cinnamate 4-hydroxylase; CHS, chalcone synthase; ROMT, resveratrol *O*-methyltransferase; CHI, chalcone isomerase; F3H, flavanone 3-hydroxylase; F3′H, flavonoid 3′-hydroxylase; F3′5′H,flavonoid 3′,5′-hydroxylase; FLS, flavonol synthase; DFR, dihydroflavonol-4-reductase; LDOX, leucoanthocyanidin dioxygenase.

**Figure 5:**
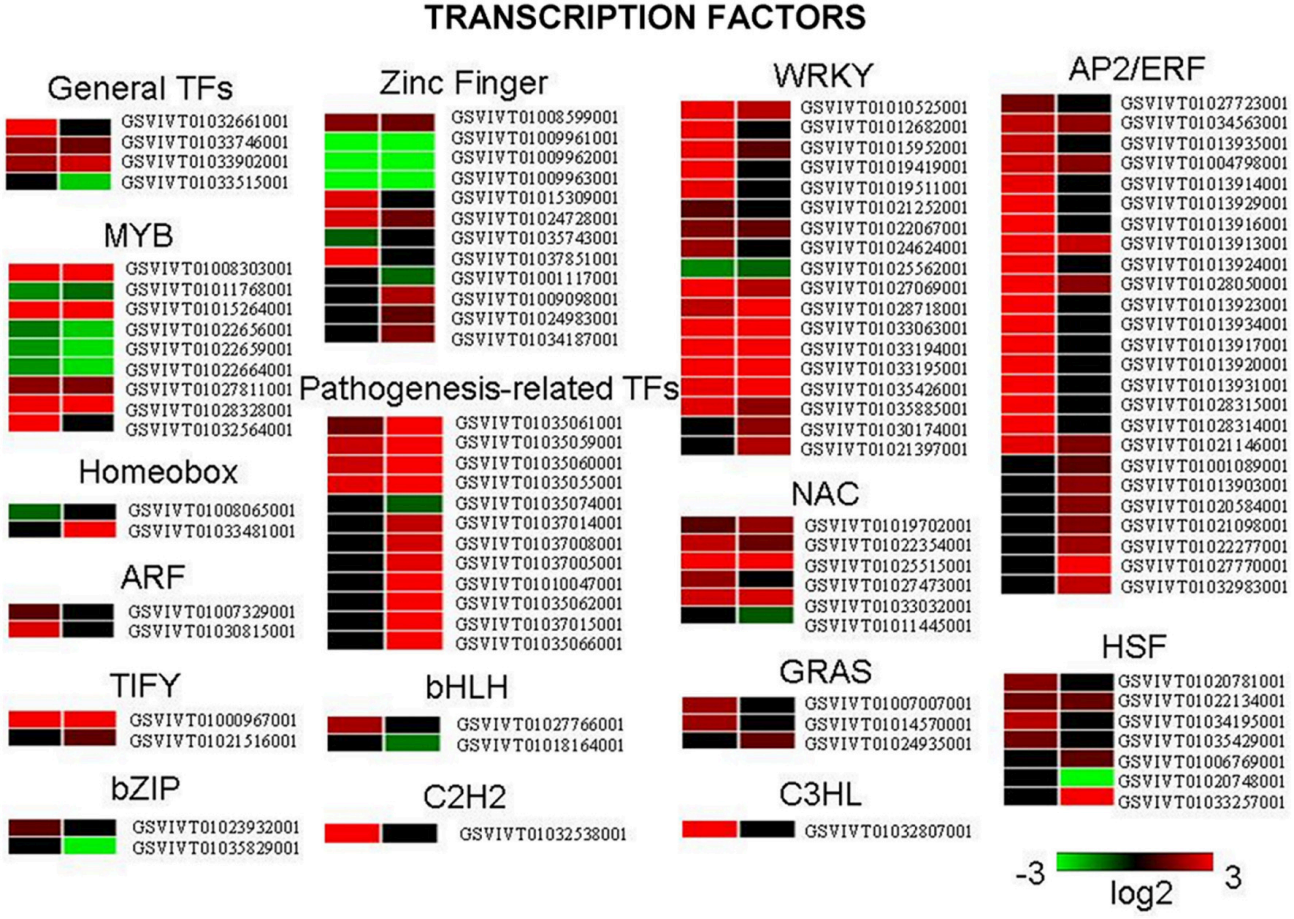
**Differentially expressed genes encoding transcription factors following UV-C treatment**. Different shades of red and green express the extent of the change according to the color bar provided (log_2_ ratio of control); black indicates no change.

Secondary metabolic pathways, such as the biosynthesis of flavonoids, phenylpropanoids (Figure 4), chlorogenic acid (Figure S5), free phenylpropanoid acid, acetyl-CoA (from pyruvate), as well as acetoin biosynthesis III and the pathway leading to the biosynthesis of phenylalanine, tyrosine, and tryptophan, all had associated genes whose expression was significantly altered 4 h and 24 h following UV-C treatment. In the case of the stilbenoid and flavonoid biosynthetic pathways, 22 genes encoding stilbene synthases (STS) exhibited 8- to 210-fold increases in their expression levels 4 h after UV-C treatment, and 29- to 64-fold increases in expression 24 h post-irradiation (Figure 4). Several genes encoding enzymes involved in phenylpropanoid biosynthesis were also highly up-regulated by the UV-C treatment (Figure 4; Tables S3, S4). These enzymes included phenylalanine ammonia-lyase (PAL) proteins, which catalyze the first step in the biosynthesis of phenylpropanoids, cinnamate 4-hydroxylase (C4H), and 4-coumarate-CoA ligases (4CL) (Figure 4). Differentially expressed UV-C responsive MYB transcription factors (transcription factors; TFs) were also identified in this study (Figure 5), with a total of 9 MYB TFs identified 4 h and 24 h following UV-C treatment, including 5 with higher and 4 with lower expression.

**Figure 6:**
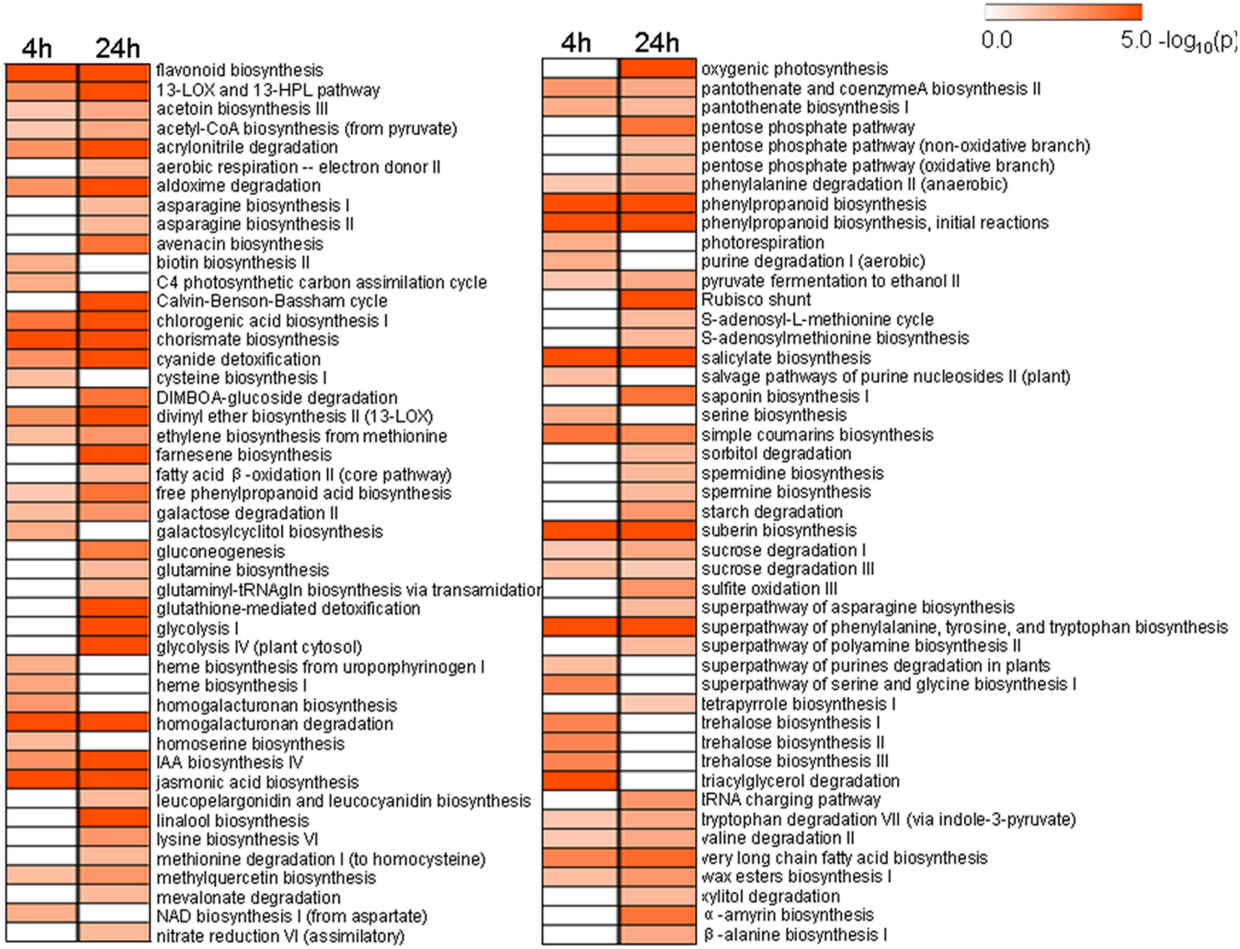
**Significantly changed pathways 4 h and 24 h after UV-C treatment with a *P*-value cutoff < 0.05**. Numbers on the color bar indicate –log (*P*-value), where the *P*-value represents the significance.

### qRT-PCR validation of DEGs identified from RNA-seq

**Figure 7:**
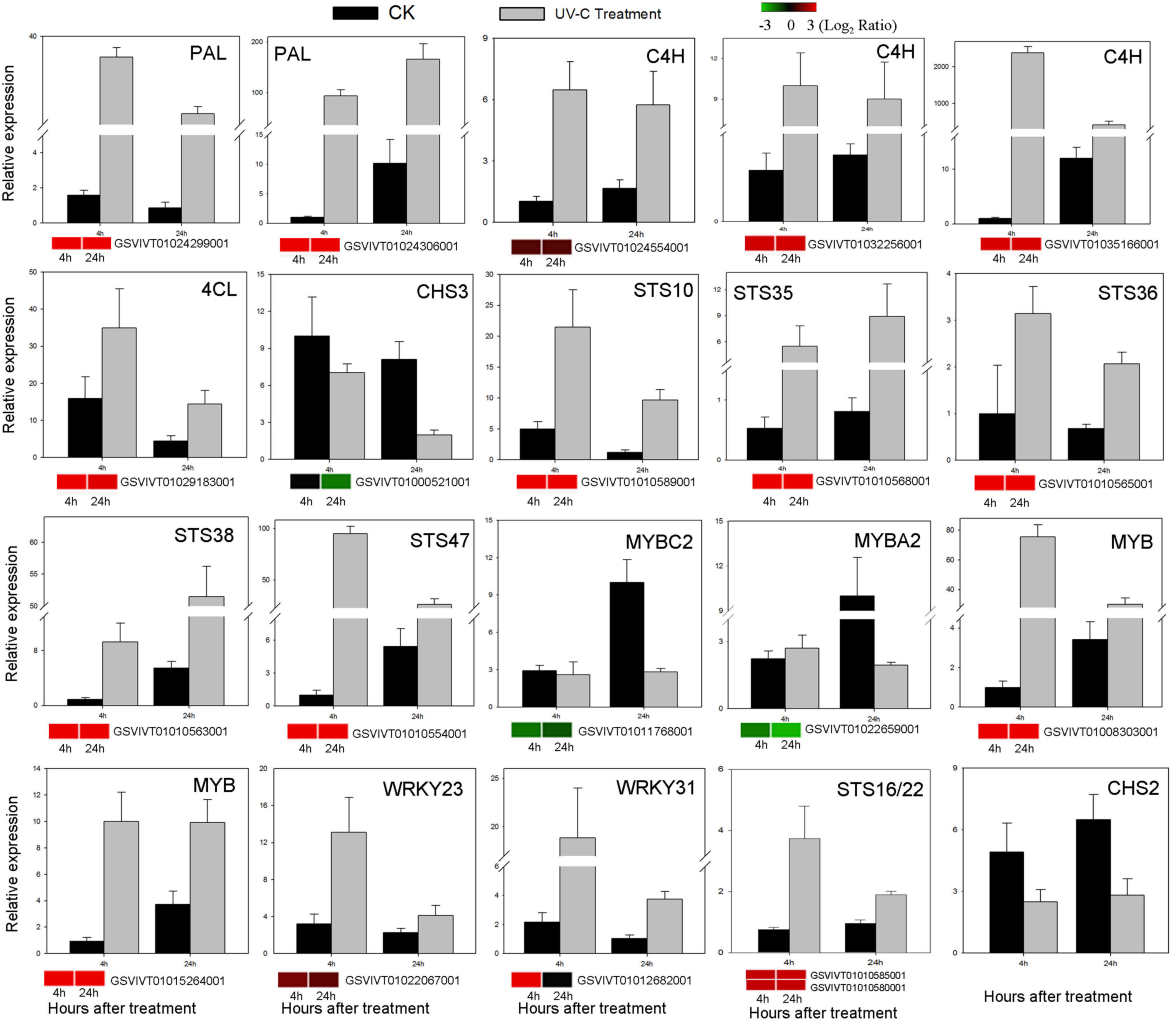
**Verification of RNA-Seq results by qRT-PCR analysis of the expression of 20 genes**. Histograms represent abundance assessed by qRT-PCR data, reported as means ± SE of two biological replicates (each biological replicate for three technical replicates). Heat maps represent changes in gene expression. The color scale represents relative expression levels, with red denoting up-regulation, green denoting down-regulation and black denoting no change.

To further validate our RNA-Seq expression profile data, we performed qRT-PCR assays of 20 selected DEGs (Figure 7 and Figure S6, Table S9). Correlations between RNA-Seq and qRT-PCR results were evaluated by comparing fold-change measurements, whereby scatterplots were generated using the log_2_ fold-changes obtained using the RNA-Seq and qRT-PCR methods (Table S10). A close correlation (*R*^2^ = 0.72) was observed between the log_2_-fold changes measured by RNA-Seq and qRT-PCR (Figure S7).

### *VaSTS* promoter analysis

To investigate potential regulatory *cis*-acting elements within a 1.5 kb region upstream (GenBank Accession number KU376402) of one of *VaSTS* genes, the sequence was analyzed using the PlantCARE database. This investigation revealed that the promoter contained typical TATA and CAAT boxes, as well as several light-regulated elements, such as a 3-AF1 binding site, G-box, AT1-motif, GAG-motif, GT1-motif, and 4cl-CMA2b element, which are homologous to *cis*-acting elements previously identified in plants. We also identified other *cis*-regulatory elements, such as a HSE motif, MBS motif, AAGAA-motif, GCN4_motif, AC-I element, Skn-1 motif and circadian element in the promoter region (Table 4). Most of the predicted motifs are known to be involved in responses to environmental stress, which suggests that *VaSTS* plays a role in defense. Based on the results of the analysis shown in Table 4, it can also be inferred that *VaSTS* may be a light-inducible gene.

**Table 4:** **Details of *cis*-acting elements involved in light- and stress-responses**.

**Name of *cis* element**	**Sequence**	**Number of *cis* elements**	**±Strand**	**Function**
3-AF1 binding site	AAGAGATATTT	2	±	Light responsive
5′UTR Py-rich stretch	TTTCTTCTCT	4	±	High transcription levels
Box I	TTTCAAA	1	+	Light responsive
CAAT-box	CAAT	36	±	Common *cis*-acting element in promoter and enhancer regions
CATT-motif	GCATTC	3	±	Part of a light responsive element
G-box	CACGAC	1	–	Light responsive
HSE	AAAAAATTTC	4	±	Heat stress responsive
MBS	CAACTG	2	±	MYB binding site
Skn-1 motif	GTCAT	1	±	*cis-acting* regulatory element required for endosperm expression
TATA-box	TATA/TTTTA/ATATAAT	68	±	Core promoter element
AAGAA-motif	GAAAGAA	1	+	Unknown
ABRE	TACGTG/AGTACGTGGC	2	+	*cis*-acting element involved in the response to abscisic acid
ACE	AAAACGTTTA	2	±	*cis*-acting element involved in light responsiveness
AT1-motif	AATTATTTTTTATT	1	–	Part of a light responsive module
Box 4	ATTAAT	3	+	Part of a conserved DNA module involved in light responsiveness
ATCT-motif	AATCTAATCT	1	+	Part of a conserved DNA module involved in light responsiveness
AC-I	TCTCACCAACC	1	–	Unknown
AC-II	CTCACCAACCCC	1	–	Element involved in negative regulation on phloem expression; and responsible for restricting expression to the xylem
CGTCA-motif	CGTCA	1	–	*cis*-acting regulatory element involved in the response to MeJA
GAG-motif	AGAGAGT	1	+	Part of a light responsive element
Gap-box	CAAATGAA(A/G)A	1	+	Part of a light responsive element
GCN4_motif	CAAGCCA	1	+	*cis*-regulatory element involved in endosperm expression
L-box	TCTCACCAACC	4	±	Part of a light responsive element
GT1-motif	GGTTAA	1	+	Light responsive element
TGACG-motif	TGACG	1	+	*cis*-acting regulatory element involved in the response to MeJA
Unnamed__4	CTCC	6	±	Unknown
Unnamed__1	CGTGG	2	+	Unknown
TCT-motif	TCTTAC	2	+	Part of a light responsive element
TCA-element	GAGAAGAATA	1	–	*cis*-acting element involved in the response to salicylic acid
circadian	CAANNNNATC	3	±	*cis*-acting regulatory element involved in circadian control

### Analysis of *VaSTS* promoter activity in response to light

**Figure 8:**
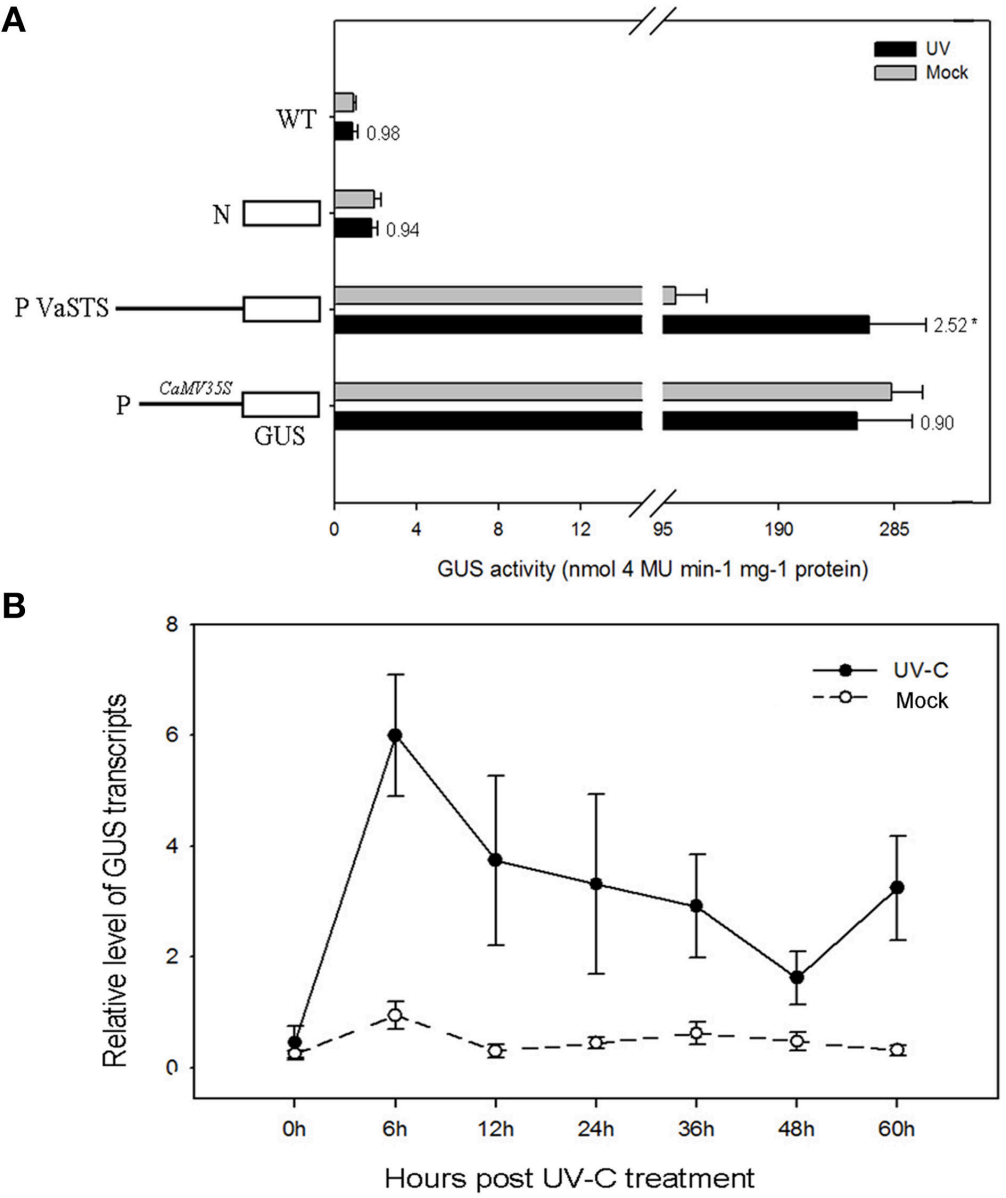
**(A)** Histochemical analysis of GUS (**β**-galactosidase) activity after UV-C treatment of transiently transformed tobacco leaves carrying the GUS coding region fused to the *VaSTS* promoter. Numbers above the bars indicate the GUS activity fold of UV-C treated GUS activity relative to that of mock-inoculated sample, and its significant difference was assessed by a one-sided paired *t*-test (*P* < 0.05 [^*^]). **(B)** Activity of the *VaSTS* promoter in tobacco at different time points following UV-C irradiation was determined by quantitative real-time RT-PCR. GUS transcript accumulation was monitored in mock-treated leaves (dashed lines) and UV-C treated leaves (solid lines). Transcript levels were expressed as relative values normalized to the transcript level of the ubiquitin gene. Results are means of triplicate experiments; error bars indicate SD.

To test the light-inducible activity of the *VaSTS* promoter, a 1570 bp fragment upstream from the transcriptional start site was fused to the *GUS* reporter gene. The construct was introduced into tobacco leaves and these were tested for GUS activity after UV-C irradiation treatment (Figure 8). A *CaMV35S*::*GUS* (pC35SGUS) construct was used as a positive control, and a promoter-less construct (pC0380GUS) served as the negative control. The response of the *VaSTS* promoter was initially analyzed by histochemical GUS staining 24 h after UV-C treatment. As shown in Figure 8A, compared with levels in untreated leaves, GUS activity was significantly greater in UV-C treated leaves. The *CaMV35S* promoter showed no significant induction after UV-C treatment, while the negative control and wild-type only exhibited low background levels of GUS activity regardless of the treatment (Figure S8).

### Verification of light-responsiveness of the *VaSTS* promoter using qRT-PCR

To further confirm that the *VaSTS* promoter contributes to the differential transcriptional regulation of expression following UV-C treatment, the promoter-*GUS* fusion constructs were used in an *Agrobacterium*-mediated transient tobacco expression system and subsequently analyzed using qRT-PCR. This data showed kinetic and dynamic changes of promoter-driven *GUS* expression in response to UV-C irradiation at the transcriptional level. Compared with levels in untreated leaves, *GUS* expression was greatly increased in UV-C treated leaves (Figure 8B). None of the wild-type tobacco leaves treated with UV-C or untreated leaves showed any detectable *GUS* expression, whereas high levels of *GUS* expression were found in light-induced and untreated leaves infiltrated with the *CaMV35S::GUS* construct. In summary, there was a low and comparable level of basal *GUS* expression in the transformed tobacco leaves at 0 h, which transiently increased in the UV-C-treated leaves at 0–6 h. A peak in *GUS* mRNA levels in transformed tobacco leaves harboring the *VaSTS* promoter constructs and treated with UV-C irradiation was observed 6 h after treatment, and thereafter *GUS* transcript levels decreased to relatively low levels. No significant change in expression was seen in the untreated plants.

## Discussion

Ultraviolet light is considered one of several significant environmental factors affecting the development and physiology of plants. While numerous studies to date have investigated the effect of UV radiation on diverse plant species, most have focused on the biosynthesis and accumulation of various metabolites in response to UV-B radiation (Brosche and Strid, 2003; Frohnmeyer and Staiger, 2003; Ulm and Nagy, 2005; Jenkins, 2009; Pontin et al., 2010). UV-C light is a germicidal type of non-ionizing radiation, and it has been widely studied for its ability to disinfect fresh fruits and vegetables to preserve their quality (Bintsis et al., 2000; Yaun et al., 2004). It has been found previously that grape berries respond to UV-C irradiation exposure by inducing the production of phytoalexins; mainly the stilbene resveratrol. However, the mechanisms underlying this UV-C-induced biosynthesis and accumulation of resveratrol are not well understood.

To advance our understanding of these responses to exogenous UV-C irradiation, we conducted an analysis of resveratrol levels in grape berries following UV-C irradiation, as well as RNA-Seq transcriptome profiling at two time points (4 and 24 h) following UV-C treatment, using untreated berries as a control. In addition, we also investigated the responsiveness of a stilbene synthase (*STS*) gene using a promoter-*GUS* fusion approach.

### UV-C irradiation causes the accumulation of stilbene compounds in grape berries

Phenolic compounds in grapes can be divided into two groups: flavonoids and non-flavonoids (Teissedre et al., 1996). Resveratrol (*trans*-3, 5, 4′-trihydroxystilbene) is a non-flavonoid polyphenol with a wide range of beneficial biological properties. To date, resveratrol has been found in at least 72 plant species, distributed among 31 genera and 12 families (Counet et al., 2006), and a number of these are components of the human diet, such as cranberry, peanut, cocoa, and especially grape and its products, such as wine. To examine the biochemical basis of stilbene accumulation in grape berries, we compared their profiles in mature fruit from various accessions following UV-C treatment, with a focus on resveratrol accumulation, and found that *V. amurensis* cv. Tonghua-3 berries showed the highest resveratrol contents. Furthermore, resveratrol levels in this accession were found to increase between 4 and 60 h post-treatment (Figure 2), which is consistent with previous findings (Douillet-Breuil et al., 1999; Cantos et al., 2001; Liu et al., 2010; Crupi et al., 2013; Wang et al., 2013a).

### General transcriptional responses induced by UV-C radiation

For each RNA-Seq sample, approximately 60% of the reads could be assigned to grape genes and were used for gene expression profiling (Table S1). There was a high correlation among biological replicates, indicating the robustness of the RNA-Seq dataset (Table S2). Moreover, a very close correlation was observed between relative expression levels based on RNA-Seq and qRT-PCR data (Figure 7; Table S10), suggesting a high level of consistency between the two analytical techniques.

We identified 881 and 1058 genes that exhibited changes in transcript abundance at 4 and 24 h post-UV-C treatment, respectively (Figure S2). Analysis of these DEGs indicated that UV-C irradiation influenced many defense and general stress response pathways, and promoted the expression of genes involved in UV-C protection or the alleviation of UV-C damage; results that are congruent with previous studies of grapevine following UV-B treatment (Pontin et al., 2010).

Increased expression of genes with a putative function in the response to biotic stimuli was a common feature of UV-C-irradiated berries at both the 4 and 24 h post-treatment time points (Tables 2, 3), which is consistent with the fact that UV-C elicited responses are known to share gene activation and signal transduction pathways commonly associated with biotic stress (Yao et al., 2012; Maharaj et al., 2014). Accordingly, several genes putatively involved in pathogen signal transduction and defense responses were also found to have higher expression in treated berries, such as those encoding NBS-LRR type disease resistance proteins (GSVIVT01018889001 and GSVIVT01030098001), an Avr9/Cf-9 rapidly elicited protein (GSVIVT01032581001) and syringolide-induced proteins (GSVIVT01022165001) (Tables S3, S4). These overlapping responses following UV-C exposure, pathogenesis and wounding stimuli may result from common signaling molecules mediating wound/ defense responses, suggesting the use of shared components, and possibly a mechanism of cross-tolerance. Thus, the effects of UV-C light on the induction of biotic stress associated-genes may improve plant resistance to pathogens and pests.

### Complex regulation of hormone signal transduction

Plant hormones play important roles in diverse growth and developmental processes, as well as in various plant responses to biotic and abiotic stress. In particular, ethylene, auxins, abscisic acid (ABA) and brassinosteroids have been associated with UV-acclimated phenotypes (Jansen, 2002; Ulm et al., 2004; Hectors et al., 2007; Berli et al., 2010), although other hormones, such as cytokinins (CTKs), gibberellins (GAs), jasmonates (JAs) and salicylic acid (SA) may also be involved (Piotrowska and Bajguz, 2011). In the present study, the expression of genes involved in the biosynthesis of JA, SA, and ethylene from methionine, were significantly altered following UV-C irradiation (Figures S9–S11; Table S8).

Genes involved in JA biosynthesis were up-regulated both 4 and 24 h after UV-C treatment (Figure S9), which correlates well with the observation that JA biosynthetic genes are responsive to various types of stress (Doornbos et al., 2011; Hind et al., 2011; Carvalhais et al., 2013; Van der Does et al., 2013). Similarly, we found that the expression of genes related to both SA and ethylene biosynthesis were up-regulated in response to UV-C treatment (Figures S10, S11; Table S8), which may result in an increase in SA and ethylene production. Both ethylene and SA are known to play key roles in biotic stress signaling in response to pathogen infection (Glazebrook, 2005), which again indicates an overlap between the various stress response pathways. Ethylene response factor (ERF) proteins, which belong to a large family of TFs that bind to the promoter of many ethylene inducible defense-related genes, are an integral component of this mechanism (Hao et al., 1998). We observed that the expression of several annotated ERF genes (GSVIVT 01034563001, GSVIVT01004798001, GSVIVT01013913001, GSVIVT01028050001 and GSVIVT01021146001) was up-regulated following UV-C treatment, both at 4 h and 24 h post-irradiation (Figure 5). In addition to its important role in pathogen defense, ethylene also plays a vital role in grape berry development and ripening, including the regulation of genes involved in anthocyanin biosynthesis and accumulation (Chervin et al., 2004). Our results therefore suggest that ethylene may also participate in the modulation of gene expression responses to UV-C radiation (Table S8 and Figure S10).

### Regulation of the expression of genes encoding TFs by UV-C irradiation

Many plant TFs are known to be rapidly induced by UV-C and likely play roles in UV-C-induced responses. In this study, we observed that the expression of TFs belonging to the AP2/EREBP family showed the most substantial changes in response to UV-C treatment, followed by MYB, WRKY, and NAC TFs (Figure 5; Tables S3, S4).

MYB genes are unique to eukaryotes (Yang et al., 2003; Jiang et al., 2004) and although their function in animals is restricted to the control of cell division and differentiation (Weston, 1998), they play important roles in plants during various physiological processes (Martin and Paz-Ares, 1997). Their expression is modulated by various hormones (Yanhui et al., 2006) and they have been shown to participate in processes related to epidermal cell fate (Ramsay and Glover, 2005), seed development (Penfield et al., 2001), responses to drought (Abe et al., 1997) and cold (Zhu et al., 2005; Agarwal et al., 2006), pathogen-disease resistance (Lee et al., 2001; Vailleau et al., 2002; Mengiste et al., 2003) and sucrose related responses (Teng et al., 2005), among other functions. Our data indicated that many MYB TFs were transcriptionally induced (between 3- and 30-fold) both 4 and 24 h post-UV-C irradiation. In particular, the gene encoding the MYB protein R2R3 Myb14 (GSVIVT01028328001), which may act as a direct activator of the *STS* genes, was found to be highly up-regulated following UV-C treatment; a result that is in agreement with previous studies (Höll et al., 2013; Fang et al., 2014; Wang et al., 2015). It should be noted that the expression of *MYB14* was not induced by UV irradiation in other studies (Pontin et al., 2010; Carbonell-Bejerano et al., 2014; Suzuki et al., 2015); a difference that may reflect the use of different cultivars, tissues, and experimental conditions, highlighting the complexity of the regulatory networks controlled by TFs.

Both NAC and WRKY TFs also play important roles in plants during development, growth and responses to environmental stress stimuli (Olsen et al., 2005b; Guo et al., 2014; Song and Nan, 2014). NAC domain (Petunia NAM and Arabidopsis ATAF1/2 and CUC2) proteins are plant-specific TFs that are expressed in various developmental stages and tissues, although details of their interactions with DNA and with other proteins are still limited (Olsen et al., 2005a,b), while WRKY proteins are known to contain a DNA binding domain bearing an invariant WRKYGQK sequence (after which the domain was named; Eulgem et al., 2000). As was the case for members of the MYB TF family, genes encoding members of the WRKY and NAC families were found to be transcriptionally induced following UV-C treatment (Figure 5), suggesting a role for these TFs in protection against UV-C radiation.

### Candidate genes that may play a role in the UV-C-induced increase in resveratrol content

It is known that the biosynthesis of secondary metabolites, such as flavonoids and other UV-C absorbing phenolic compounds, which accumulate in the vacuoles of epidermal cells, is an important factor in UV-C acclimation. Grapevine plantlets exposed to UV-C showed an up-regulation of genes annotated as being involved in secondary metabolism, especially those associated with the phenylpropanoid pathway (Figure 6; Table S8). Indeed, most *PAL* genes, as well as genes encoding C4H and 4CL, were up-regulated in response to UV-C treatment (Figure 4).

In addition, the resveratrol producing pathway in grape is known to be dependent upon the function of *STS* genes. In this study, we found 22 *STS* genes which were actively responsive to UV-C irradiation in grape, with up-regulation occurring in every case. In grapevine, chalcone synthase (CHS) substrates can also be used by STS enzymes for the production of stilbenes, which are classified as phytoalexins because of their role in plant defense mechanisms against fungal pathogens (Langcake and Pryce, 1977; Jeandet et al., 2002). Interestingly, we found that *CHS* genes (GSVIVT01000521001 and GSVIVT01032968001), which are also involved in flavonoid biosynthesis, were down-regulated after UV-C irradiation (Figure 4). In agreement with our results for the expression of genes encoding STS and CHS (Figure 4; Tables S3, S4), it has previously been found that *CHS* genes were down-regulated and genes encoding STS were up-regulated after UV-C treatment (Xi et al., 2014; Suzuki et al., 2015). These results suggest that since STS and CHS share a substrate, there may be a competitive and/or inhibitory relationship between them in response to UV-C exposure, which may in turn play a role in resveratrol accumulation in grape berries.

### The activity of the *VaSTS* promoter is regulated by UV irradiation and may contribute to the accumulation of resveratrol

Previous studies have shown that the promoters of *STS* genes contain several important elements, including Box-W1 and MBS motifs (Xu et al., 2011). Box-W1 (TTGACC), which is similar to the conserved core sequence of the fungal elicitor-responsive element found in *Petroselinum crispum*, is specifically recognized by a salicylic acid-induced WRKY DNA-binding protein (Rushton et al., 1996), while the MBS element is a binding consensus site for a MYB homolog (Urao et al., 1993). As such, it is possible that specific WRKY and/or MYB TFs that are up-regulated following UV-C treatment may lead to positive regulation of *STS* genes in grape. However, another possibility, which is not mutually exclusive, is that UV-C radiation is able to stimulate certain *STS* promoter elements directly through light-responsive elements (Table 4).

In this study, we utilized a *VaSTS* promoter-*GUS* fusion construct and transient expression assays in tobacco to determine whether the UV-responsiveness of this gene was due to elements within its promoter. Quantitative real-time RT-PCR data of *GUS* expression patterns (Figure 8B) and GUS activity data (Figure 8A) suggested that this promoter exhibited significantly higher levels of activity when leaves were exposed to UV-C irradiation. This is in agreement with previous findings in which a grapevine *STS* promoter isolated from *V. vinifera* cv. *Optima* was shown to be activated in response to biotic (Liswidowati et al., 1991; Bais et al., 2000; Keller et al., 2003) and abiotic stresses, such as wounding (Jeandet et al., 1997). In addition, our *in silico* analysis indicated that the *VaSTS* promoter contained several light-responsive elements, including a GAG-motif, which is part of the rbcA conserved DNA module array (rbcA-CMA1) involved in light responsiveness, and an L-box, which is a *cis*-acting regulatory element associated with the A- and P-box (Logemann et al., 1995). These results, along with our UV-C treatment data, indicate that the expression of the *VaSTS* gene is regulated via motifs in its promoter that are responsive to UV-C light irradiation.

In conclusion, our transcriptome analysis revealed that UV-C irradiation had regulatory effects on defense and general stress response pathways, as well as on genes involved in UV-C protection or the alleviation of UV-C damage, which likely contributes to the acclimation of plants to UV-C exposure. We have also identified several potential structural and regulatory genes, as well as other factors, which may play a role in the accumulation of resveratrol in grape berries following UV-C exposure. These results provide an important framework for the further elucidation of precise mechanism by which resveratrol biosynthesis is induced by UV-C and other types of stress.

## Availability of supporting data

The datasets supporting the results of this article have been submitted to the Sequence Read Archive at NCBI (http://www.ncbi.nlm.nih.gov/sra). The submission code is PRJNA286165.

## Author contributions

XW, XY: conceived and designed the experiments. XY, CJ, YL, and ZF: analyzed the data. XY, HW, HQ: performed the experiments. XW, YW, and ZL: contributed reagents/materials/analysis tools. XW, CF: provided guidance for the entire study. XY, SS, and XW: wrote the manuscript. All authors approved the final manuscript.

### Conflict of interest statement

The authors declare that the research was conducted in the absence of any commercial or financial relationships that could be construed as a potential conflict of interest.

## Supplementary material

Table S1 **Number of reads cleaned and mapped with Tophat following UV-C irradiation of Tonghua-3 mature grape berries**. 4h-T1, 4h-T2, 24h-T1, 24h-T2: treated samples 4 and 24 h following UV-C irradiation; 4h-CK1, 4h-CK2, 24h-CK1, 24h-CK2: untreated samples at 4 and 24 h.

Table S2 **Correlation coefficients of transcriptome profiles among the RNA-Seq samples from Tonghua-3 mature grape berries**. 4h-T1, 4h-T2, 24h-T1, 24h-T2: treated samples 4 and 24 h post-irradiation; 4h-CK1, 4h-CK2, 24h-CK1, 24h-CK2: untreated samples at 4 and 24 h.

Table S3 **Full list of differentially expressed genes 4 h following UV-C radiation**. Includes grapevine gene ID, GenBank top hit accession number, annotation and fold-change compared with untreated control samples.

Table S4 **Full list of differentially expressed genes 24 h following UV-C radiation**. Includes grapevine gene ID, GenBank top hit accession number, annotation and fold-change compared with untreated control samples.

Table S5 **Functional categorization of differentially expressed genes after UV-C treatment within the “biological process” category of Gene Ontology (GO)**.

Table S6 **Functional categorization of differentially expressed genes after UV-C treatment within the “molecular function” category of Gene Ontology (GO)**.

Table S7 **Functional categorization of differentially expressed genes after UV-C treatment within the cellular component category of Gene Ontology (GO)**.

Table S8 **Significantly altered pathways 4 and 24 h after UV-C treatment with a *P*-value cutoff of < 0.05**.

Table S9 **Primer sequences used for qRT-PCR**.

Table S10 **Experimental validation of RNA-Seq results using qRT-PCR expression analysis**.

Figure S1 **Overview of the experimental design in this study**.

Figure S2 **Venn diagrams showing the distribution of RNA-Seq data**.

Figure S3 **Functional categorization of differentially expressed genes after UV-C treatment within the “molecular function” category of Gene Ontology (GO)**.

Figure S4 **Functional categorization of differentially expressed genes after UV-C treatment within the “cellular component” category of Gene Ontology (GO)**.

Figure S5 **Changes in transcript levels of genes involved in chorismate biosynthesis following UV-C treatment**. Different shades of red and green show the extent of the change according to the color bar provided (log2 ratio of control); black indicates no change; gray indicates no transcript detected.

Figure S6 **Verification of RNA-Seq results by qRT-PCR analysis of the expression of 20 genes**. The grape *GAPDH* gene (GenBank accession CB973647) was used as the reference gene. Histograms represent abundance assessed by qRT-PCR data, reported as means ± SE of two biological replicates (each biological replicate comprised three technical replicates). Heat maps represent changes in gene expression. The color scale represents relative expression levels, with red denoting up-regulation, green denoting down-regulation and black denoting no change.

Figure S7 **Comparison of the log2 (fold-change; FC) of 20 selected transcripts using RNA-Seq and qRT-PCR analyses**.

Figure S8 **Histochemical assay for GUS expression in transiently transformed leaves of *Nicotiana benthamiana* after UV-C treatment**. Fully expanded leaves from 6 week old *N. benthamiana* plants were agro-infiltrated with the *VaSTS* promoter construct at a concentration of OD600 0.6. WT wild type, N represents negative control (pC0380GUS), P represents positive control (pC35SGUS).

Figure S9 **Changes in transcript levels of genes involved in jasmonic acid synthesis following UV-C treatment**. Different shades of red and green show the extent of the change according to the color bar provided (log2 ratio of control); black indicates no change; gray indicates no transcript detected.

Figure S10 **Changes in transcript levels of genes involved in salicylate biosynthesis following UV-C treatment**. Different shades of red and green express the extent of the change according to the color bar provided (log2 ratio of control); black indicates no change; gray indicates no transcript detected.

Figure S11 **Changes in transcript levels of genes involved in ethylene biosynthesis from methionine following UV-C treatment**. Different shades of red and green show the extent of the change according to the color bar provided (log2 ratio of control); black indicates no change; gray indicates no transcript detected.

## Abbreviations

UV-C: Ultraviolet-C radiation
DEG: differentially expressed gene
RPKM: reads per kilobase of exon model per million mapped reads
FDR: false discovery rate
GO: Gene Ontology
GA: gibberellic acid
JA: jasmonic acid
ABA: abscisic acid
CTK: cytokinin
SA: salicylic acid
PAL: phenylalanine ammonia-lyase
C4H: cinnamate 4-hydroxylase
4CL: 4-coumarate-CoA ligase
STS: stilbene synthase
CHS: chalcone synthase
qRT-PCR: quantitative real-time-PCR
TF: transcription factor
ERF: ethylene response factor.

## References

[B1] Abe H. YamaguchiShinozaki K. Urao T. Iwasaki T. Hosokawa D. Shinozaki K. (1997). Role of Arabidopsis MYC and MYB homologs in drought- and abscisic acid-regulated gene expression. Plant Cell 9, 1859–1868. 9368419 10.1105/tpc.9.10.1859 PMC157027

[B2] Agarwal M. Hao Y. J. Kapoor A. Dong C. H. Fujii H. Zheng X.. (2006). A R2R3 type MYB transcription factor is involved in the cold regulation of CBF genes and in acquired freezing tolerance. J. Biol. Chem. 281, 37636–37645. 10.1074/jbc.M605895200 17015446

[B3] Anders S. Huber W. (2010). Differential expression analysis for sequence count data. Genome Biol. 11:R106. 10.1186/gb-2010-11-10-r106 20979621 PMC3218662

[B4] Bais A. J. Murphy P. J. Dry I. B. (2000). The molecular regulation of stilbene phytoalexin biosynthesis in *Vitis vinifera* during grape berry development. Funct. Plant Biol. 27, 723–723. 10.1071/PP00007

[B5] Baur J. A. Sinclair D. A. (2006). Therapeutic potential of resveratrol: the *in vivo* evidence. Nat. Rev. Drug Discov. 5, 493–506. 10.1038/nrd2060 16732220

[B6] Benjamini Y. Hochberg Y. (1995). Controlling the false discovery rate: a practical and powerful approach to multiple testing. J. R. Stat. Soc. B Methodol. 57, 289–300.

[B7] Berli F. D'Angelo J. Cavagnaro B. Bottini R. Wuilloud R. Silva M. F. (2008). Phenolic composition in grape (*Vitis vinifera* L. cv. Malbec) ripened with different solar UV-B radiation levels by capillary zone electrophoresis. J. Agric. Food Chem. 56, 2892–2898. 10.1021/jf073421+ 18412357

[B8] Berli F. J. Moreno D. Piccoli P. Hespanhol-Viana L. Silva M. F. Bressan-Smith R.. (2010). Abscisic acid is involved in the response of grape (*Vitis vinifera* L.) cv. Malbec leaf tissues to ultraviolet-B radiation by enhancing ultraviolet-absorbing compounds, antioxidant enzymes and membrane sterols. Plant Cell Environ. 33, 1–10. 10.1111/j.1365-3040.2009.02044.x 19781012

[B9] Bintsis T. Litopoulou-Tzanetaki E. Robinson R. K. (2000). Existing and potential applications of ultraviolet light in the food industry - a critical review. J. Sci. Food Agric. 80, 637–645. 10.1002/(SICI)1097-0010(20000501)80:6<637::AID-JSFA603>3.0.CO;2-1 29345786

[B10] Bradford M. M. (1976). A rapid and sensitive method for the quantitation of microgram quantities of protein utilizing the principle of protein-dye binding. Anal. Biochem. 72, 248–254. 10.1016/0003-2697(76)90527-3 942051

[B11] Brosche M. Strid A. (2003). Molecular events following perception of ultraviolet-B radiation by plants. Physiol. Plant. 117, 1–10. 10.1034/j.1399-3054.2003.1170101.x

[B12] Cantos E. Espín J. C. Tomás-Barberán F. A. (2001). Postharvest induction modeling method using UV irradiation pulses for obtaining resveratrol-enriched table grapes: a new “functional” fruit? J. Agric. Food Chem. 49, 5052–5058. 10.1021/jf010366a 11600065

[B13] Carbonell-Bejerano P. Diago M. P. Martínez-Abaigar J. Martínez-Zapater J. M. Tardáguila J. Núñez-Olivera E. (2014). Solar ultraviolet radiation is necessary to enhance grapevine fruit ripening transcriptional and phenolic responses. BMC Plant Biol. 14:183. 10.1186/1471-2229-14-183 25012688 PMC4099137

[B14] Carvalhais L. C. Dennis P. G. Badri D. V. Tyson G. W. Vivanco J. M. Schenk P. M. (2013). Activation of the jasmonic acid plant defence pathway alters the composition of rhizosphere bacterial communities. PLoS ONE 8:e56457. 10.1371/journal.pone.0056457 23424661 PMC3570460

[B15] Chervin C. El-Kereamy A. Roustan J. P. Latche A. Lamon J. Bouzayen M. (2004). Ethylene seems required for the berry development and ripening in grape, a non-climacteric fruit. Plant Sci. 167, 1301–1305. 10.1016/j.plantsci.2004.06.026

[B16] Chong J. L. Poutaraud A. Hugueney P. (2009). Metabolism and roles of stilbenes in plants. Plant Sci. 177, 143–155. 10.1016/j.plantsci.2009.05.012

[B17] Counet C. Callemien D. Collin S. (2006). Chocolate and cocoa: new sources of trans-resveratrol and trans-piceid. Food Chem. 98, 649–657. 10.1016/j.foodchem.2005.06.030

[B18] Crupi P. Pichierri A. Basile T. Antonacci D. (2013). Postharvest stilbenes and flavonoids enrichment of table grape cv Redglobe (*Vitis vinifera* L.) as affected by interactive UV-C exposure and storage conditions. Food Chem. 141, 802–808. 10.1016/j.foodchem.2013.03.055 23790850

[B19] Doornbos R. F. Geraats B. P. J. Kuramae E. E. Van Loon L. C. Bakker P. A. H. M. (2011). Effects of jasmonic acid, ethylene, and salicylic acid signaling on the rhizosphere bacterial community of *Arabidopsis thaliana*. Mol. Plant Microbe Interact. 24, 395–407. 10.1094/MPMI-05-10-0115 21171889

[B20] Douillet-Breuil A. C. Jeandet P. Adrian M. Bessis N. (1999). Changes in the phytoalexin content of various Vitis spp. in response to ultraviolet C elicitation. J. Agric. Food Chem. 47, 4456–4461. 10.1021/jf9900478 10552833

[B21] Eisen M. B. Spellman P. T. Brown P. O. Botstein D. (1998). Cluster analysis and display of genome-wide expression patterns. Proc. Natl. Acad. Sci. U.S.A. 95, 14863–14868. 10.1073/pnas.95.25.14863 9843981 PMC24541

[B22] Eulgem T. Rushton P. J. Robatzek S. Somssich I. E. (2000). The WRKY superfamily of plant transcription factors. Trends Plant Sci. 5, 199–206. 10.1016/S1360-1385(00)01600-9 10785665

[B23] Fang L. Hou Y. Wang L. Xin H. Wang N. Li S. (2014). Myb14, a direct activator of STS, is associated with resveratrol content variation in berry skin in two grape cultivars. Plant Cell Rep. 33, 1629–1640. 10.1007/s00299-014-1642-3 24948530

[B24] Floratos A. Smith K. Ji Z. Watkinson J. Califano A. (2010). geWorkbench: an open source platform for integrative genomics. Bioinformatics 26, 1779–1780. 10.1093/bioinformatics/btq282 20511363 PMC2894520

[B25] Frohnmeyer H. Staiger D. (2003). Ultraviolet-B radiation-mediated responses in plants. Balancing damage and protection. Plant Physiol. 133, 1420–1428. 10.1104/pp.103.030049 14681524 PMC1540342

[B26] Glazebrook J. (2005). Contrasting mechanisms of defense against biotrophic and necrotrophic pathogens. Annual Review of Phytopathology 43, 205–227. 10.1146/annurev.phyto.43.040204.135923 16078883

[B27] Guo C. L. Guo R. R. Xu X. Z. Gao M. Li X. Q. Song J. Y.. (2014). Evolution and expression analysis of the grape (*Vitis vinifera* L.) WRKY gene family. J. Exp. Bot. 65, 1513–1528. 10.1093/jxb/eru007 24510937 PMC3967086

[B28] Hao D. Y. Ohme-Takagi M. Sarai A. (1998). Unique mode of GCC box recognition by the DNA-binding domain of ethylene-responsive element-binding factor (ERF domain) in plant. J. Biol. Chem. 273, 26857–26861. 10.1074/jbc.273.41.26857 9756931

[B29] Hectors K. Prinsen E. De Coen W. Jansen M. A. K. Guisez Y. (2007). *Arabidopsis thaliana* plants acclimated to low dose rates of ultraviolet B radiation show specific changes in morphology and gene expression in the absence of stress symptoms. New Phytol. 175, 255–270. 10.1111/j.1469-8137.2007.02092.x 17587374

[B30] Hind S. R. Pulliam S. E. Veronese P. Shantharaj D. Nazir A. Jacobs N. S.. (2011). The COP9 signalosome controls jasmonic acid synthesis and plant responses to herbivory and pathogens. Plant J. 65, 480–491. 10.1111/j.1365-313X.2010.04437.x 21265900

[B31] Höll J. Vannozzi A. Czemmel S. D'Onofrio C. Walker A. R. Rausch T.. (2013). The R2R3-MYB transcription factors MYB14 and MYB15 regulate stilbene biosynthesis in *Vitis vinifera*. Plant Cell 25, 4135–4149. 10.1105/tpc.113.117127 24151295 PMC3877794

[B32] Hollósy F. (2002). Effects of ultraviolet radiation on plant cells. Micron 33, 179–197. 10.1016/S0968-4328(01)00011-7 11567887

[B33] Jaillon O. Aury J. M. Noel B. Policriti A. Clepet C. Casagrande A.. (2007). The grapevine genome sequence suggests ancestral hexaploidization in major angiosperm phyla. Nature 449, U463–U465. 10.1038/nature06148 17721507

[B34] Jansen M. A. K. (2002). Ultraviolet-B radiation effects on plants: induction of morphogenic responses. Physiol. Plant. 116, 423–429. 10.1034/j.1399-3054.2002.1160319.x

[B35] Jeandet P. Breuil A. C. Adrian M. Weston L. A. Debord S. Meunier P. (1997). HPLC analysis of grapevine phytoalexins coupling photodiode array detection and fluorometry. Anal. Chem. 69, 5172–5177. 10.1021/ac970582b

[B36] Jeandet P. Douillet-Breuil A.-C. Bessis R. Debord S. Sbaghi M. Adrian M. (2002). Phytoalexins from the Vitaceae: biosynthesis, phytoalexin gene expression in transgenic plants, antifungal activity, and metabolism. J. Agric. Food Chem. 50, 2731–2741. 10.1021/jf011429s 11982391

[B37] Jefferson R. A. (1987). Assaying chimeric genes in plants: the GUS gene fusion system. Plant Mol. Biol. Rep. 5, 387–405. 10.1007/BF02667740

[B38] Jenkins G. I. (2009). Signal transduction in responses to UV-B radiation. Annu. Rev. Plant Biol. 60, 407–431. 10.1146/annurev.arplant.59.032607.092953 19400728

[B39] Jiang C. Gu J. Chopra S. Gu X. Peterson T. (2004). Ordered origin of the typical two and three repeat Myb genes. Gene 326, 13–22. 10.1016/j.gene.2003.09.049 14729259

[B40] Joung J. G. Corbett A. M. Fellman S. M. Tieman D. M. Klee H. J. Giovannoni J. J.. (2009). Plant MetGenMAP: an integrative analysis system for plant systems biology. Plant Physiol. 151, 1758–1768. 10.1104/pp.109.145169 19819981 PMC2786002

[B41] Kasim M. U. Kasim R. Erkal S. (2008). UV-C treatments on fresh-cut green onions enhanced antioxidant activity, maintained green color and controlled ‘telescoping’. J. Food Agric. Environ. 6, 63–67.

[B42] Keller M. Viret O. Cole F. M. (2003). *Botrytis cinerea* infection in grape flowers: defense reaction, latency, and disease expression. Phytopathology 93, 316–322. 10.1094/PHYTO.2003.93.3.316 18944341

[B43] Langcake P. Pryce R. (1977). A new class of phytoalexins from grapevines. Experientia 33, 151–152. 10.1007/BF02124034 844529

[B44] Langmead B. Trapnell C. Pop M. Salzberg S. L. (2009). Ultrafast and memory-efficient alignment of short DNA sequences to the human genome. Genome Biol. 10:R25. 10.1186/gb-2009-10-3-r25 19261174 PMC2690996

[B45] Lee M. W. Qi M. Yang Y. O. (2001). A novel jasmonic acid-inducible rice myb gene associates with fungal infection and host cell death. Mol. Plant Microbe Interact. 14, 527–535. 10.1094/MPMI.2001.14.4.527 11310740

[B46] Lescot M, Déhais, P. Thijs G. Marchal K. Moreau Y. Van de Peer Y.. (2002). PlantCARE, a database of plant cis-acting regulatory elements a portal to tools for *in silico* analysis of promoter sequences. Nucleic Acid Res. 30, 325–327. Available online at: http://nar.oxfordjournals.org/content/30/1/325.abstract 11752327 10.1093/nar/30.1.325 PMC99092

[B47] Li X. Zheng X. Yan S. Li S. (2008). Effects of salicylic acid (SA), ultraviolet radiation (UV-B and UV-C) on trans-resveratrol inducement in the skin of harvested grape berries. Front. Agric. China 2, 77–81. 10.1007/s11703-008-0014-6

[B48] Liswidowati Melchior, F. Hohmann F. Schwer B. Kindl H. (1991). Induction of stilbene synthase by *Botrtytis cinerea* in cultured grapevine cells. Planta 183, 307–314. 10.1007/BF00197803 24193635

[B49] Liu W. Liu C. Y. Yang C. X. Wang L. J. Li S. H. (2010). Effect of grape genotype and tissue type on callus growth and production of resveratrols and their piceids after UV-C irradiation. Food Chem. 122, 475–481. 10.1016/j.foodchem.2010.03.055

[B50] Logemann E. Parniske M. Hahlbrock K. (1995). Modes of expression and common structural features of the complete phenylalanine ammonia-lyase gene family in Parsley. Proc. Natl. Acad. Sci. U.S.A. 92, 5905–5909. 10.1073/pnas.92.13.5905 7597051 PMC41610

[B51] Maharaj R. Arul J. Nadeau P. (2014). UV-C irradiation effects on levels of enzymic and non-enzymic phytochemicals in tomato. Innov. Food Sci. Emerg. Technol. 21, 99–106. 10.1016/j.ifset.2013.10.001

[B52] Martin C. Paz-Ares J. (1997). MYB transcription factors in plants. Trends Genet. 13, 67–73. 10.1016/S0168-9525(96)10049-4 9055608

[B53] Mengiste T. Chen X. Salmeron J. Dietrich R. (2003). The BOTRYTIS SUSCEPTIBLE1 gene encodes an R2R3MYB transcription factor protein that is required for biotic and abiotic stress responses in Arabidopsis. Plant Cell 15, 2551–2565. 10.1105/tpc.014167 14555693 PMC280560

[B54] Olsen A. N. Ernst H. A. Lo Leggio L. Skriver K. (2005a). DNA-binding specificity and molecular functions of NAC transcription factors. Plant Sci. 169, 785–797. 10.1016/j.plantsci.2005.05.035

[B55] Olsen A. N. Ernst H. A. Lo Leggio L. Skriver K. (2005b). NAC transcription factors: structurally distinct, functionally diverse. Trends Plant Sci. 10, 79–87. 10.1016/j.tplants.2004.12.010 15708345

[B56] Penfield S. Meissner R. C. Shoue D. A. Carpita N. C. Bevan M. W. (2001). MYB61 is required for mucilage deposition and extrusion in the Arabidopsis seed coat. Plant Cell 13, 2777–2791. 10.1105/tpc.13.12.2777 11752387 PMC139488

[B57] Piotrowska A. Bajguz A. (2011). Conjugates of abscisic acid, brassinosteroids, ethylene, gibberellins, and jasmonates. Phytochemistry 72, 2097–2112. 10.1016/j.phytochem.2011.08.012 21880337

[B58] Pontin M. A. Piccoli P. N. Francisco R. Bottini R. Martinez-Zapater J. M. Lijavetzky D. (2010). Transcriptome changes in grapevine (*Vitis vinifera* L.) cv. Malbec leaves induced by ultraviolet-B radiation. BMC Plant Biol. 10:224. 10.1186/1471-2229-10-224 20959019 PMC3017828

[B59] Ramsay N. A. Glover B. J. (2005). MYB-bHLH-WD40 protein complex and the evolution of cellular diversity. Trends Plant Sci. 10, 63–70. 10.1016/j.tplants.2004.12.011 15708343

[B60] Rushton P. J. Torres J. T. Parniske M. Wernert P. Hahlbrock K. Somssich I. E. (1996). Interaction of elicitor-induced DNA-binding proteins with elicitor response elements in the promoters of parsley PR1 genes. Embo J. 15, 5690–5700. 8896462 PMC452313

[B61] Siemann E. H. Creasy L. L. (1992). Concentration of the phytoalexin resveratrol in wine. Am. J. Enol. Vitic. 43, 49–52.

[B62] Song H. Nan Z. (2014). Genome-wide identification and analysis of WRKY transcription factors in *Medicago truncatula*. Yi Chuan 36, 152–168. 10.3724/SP.J.1005.2014.00152 24846944

[B63] Sparkes I. A. Runions J. Kearns A. Hawes C. (2006). Rapid, transient expression of fluorescent fusion proteins in tobacco plants and generation of stably transformed plants. Nat. Protoc. 1, 2019–2025. 10.1038/nprot.2006.286 17487191

[B64] Sun B. Ribes A. M. Leandro M. C. Belchior A. P. Spranger M. I. (2006). Stilbenes: quantitative extraction from grape skins, contribution of grape solids to wine and variation during wine maturation. Anal. Chim. Acta 563, 382–390. 10.1016/j.aca.2005.12.002

[B65] Suzuki M. Nakabayashi R. Ogata Y. Sakurai N. Tokimatsu T. Goto S. (2015). Multi omics in grape berry skin revealed specific induction of stilbene synthetic pathway by UV-C irradiation. Plant Physiol. 168, 47–59. 10.1104/pp.114.254375 25761715 PMC4424009

[B66] Teissedre P. Waterhouse A. Walzem R. German J. Frankel E. Ebeler S. (1996). Composés phénoliques du raisin et du vin et santé. Bull. l'OIV 69, 251–277.

[B67] Teng S. Keurentjes J. Bentsink L. Koornneef M. Smeekens S. (2005). Sucrose-specific induction of anthocyanin biosynthesis in Arabidopsis requires the MYB75/PAP1 gene. Plant Physiol. 139, 1840–1852. 10.1104/pp.105.066688 16299184 PMC1310563

[B68] Trapnell C. Pachter L. Salzberg S. L. (2009). TopHat: discovering splice junctions with RNA-Seq. Bioinformatics 25, 1105–1111. 10.1093/bioinformatics/btp120 19289445 PMC2672628

[B69] Ulm R. Baumann A. Oravecz A. Máté Z. Adám E. Oakeley E. J.. (2004). Genome-wide analysis of gene expression reveals function of the bZIP transcription factor HY5 in the UV-B response of Arabidopsis. Proc. Natl. Acad. Sci. U.S.A. 101, 1397–1402. 10.1073/pnas.0308044100 14739338 PMC337064

[B70] Ulm R. Nagy F. (2005). Signalling and gene regulation in response to ultraviolet light. Curr. Opin. Plant Biol. 8, 477–482. 10.1016/j.pbi.2005.07.004 16039155

[B71] Urao T. Yamaguchishinozaki K. Urao S. Shinozaki K. (1993). An Arabidopsis Myb homolog is induced by dehydration stress and its gene-product binds to the conserved Myb recognition sequence. Plant Cell 5, 1529–1539. 10.1105/tpc.5.11.1529 8312738 PMC160383

[B72] Vailleau F. Daniel X. Tronchet M. Montillet J. L. Triantaphylidès C. Roby D. (2002). A R2R3-MYB gene, AtMYB30, acts as a positive regulator of the hypersensitive cell death program in plants in response to pathogen attack. Proc. Natl. Acad. Sci. U.S.A. 99, 10179–10184. 10.1073/pnas.152047199 12119395 PMC126644

[B73] Van der Does D. Leon-Reyes A. Koornneef A. Van Verk M. C. Rodenburg N. Pauwels L.. (2013). Salicylic acid suppresses jasmonic acid signaling downstream of SCFCOI1-JAZ by targeting GCC promoter motifs via transcription factor ORA59. Plant Cell 25, 744–761. 10.1105/tpc.112.108548 23435661 PMC3608790

[B74] Wang J.-F. Ma L. Xi H.-F. Wang L.-J. Li S.-H. (2015). Resveratrol synthesis under natural conditions and after UV-C irradiation in berry skin is associated with berry development stages in ‘Beihong’ (*V. vinifera* × *V. amurensis*). Food Chem. 168c, 430–438. 10.1016/j.foodchem.2014.07.025 25172731

[B75] Wang L. J. Ma L. Xi H. F. Duan W. Wang J. F. Li S. H. (2013a). Individual and combined effects of CaCl2 and UV-C on the biosynthesis of resveratrols in grape leaves and berry skins. J. Agric. Food Chem. 61, 7135–7141. 10.1021/jf401220m 23855433

[B76] Wang L. J. Xu M. Liu C. Y. Wang J. F. Xi H. F. Wu B. H.. (2013b). Resveratrols in grape berry skins and leaves in Vitis germplasm. PLoS ONE 8:e61642. 10.1371/journal.pone.0061642 23637874 PMC3634843

[B77] Weston K. (1998). Myb proteins in life, death and differentiation. Curr. Opin. Genet. Dev. 8, 76–81. 10.1016/S0959-437X(98)80065-8 9529609

[B78] Xi H. Ma L. Liu G. Wang N. Wang J. Wang L.. (2014). Transcriptomic analysis of grape (*Vitis vinifera* L.) leaves after exposure to ultraviolet C irradiation. PLoS ONE 9:e113772. 10.1371/journal.pone.0113772 25464056 PMC4252036

[B79] Xu W. Yu Y. Ding J. Hua Z. Wang Y. (2010). Characterization of a novel stilbene synthase promoter involved in pathogen-and stress-inducible expression from Chinese wild *Vitis pseudoreticulata*. Planta 231, 475–487. 10.1007/s00425-009-1062-8 19937257

[B80] Xu W. R. Yu Y. H. Zhou Q. Ding J. H. Dai L. M. Xie X. Q.. (2011). Expression pattern, genomic structure, and promoter analysis of the gene encoding stilbene synthase from Chinese wild *Vitis pseudoreticulata*. J. Exp. Bot. 62, 2745–2761. 10.1093/jxb/erq447 21504880

[B81] Yang T. Perasso R. Baroin-Tourancheau A. (2003). Myb genes in ciliates: a common origin with the myb protooncogene? Protist 154, 229–238. 10.1078/143446103322166527 13677450

[B82] Yanhui C. Xiaoyuan Y. Kun H. Meihua L. Jigang L. Zhaofeng G.. (2006). The MYB transcription factor superfamily of arabidopsis: expression analysis and phylogenetic comparison with the rice MYB family. Plant Mol. Biol. 60, 107–124. 10.1007/s11103-005-2910-y 16463103

[B83] Yao Y. Danna C. H. Ausubel F. M. Kovalchuk I. (2012). Perception of volatiles produced by UVC-irradiated plants alters the response to viral infection in naïve neighboring plants. Plant Signal. Behav. 7, 741–745. 10.4161/psb.20406 22751319 PMC3583953

[B84] Yaun B. R. Sumner S. S. Eifert J. D. Marcy J. E. (2004). Inhibition of pathogens on fresh produce by ultraviolet energy. Int. J. Food Microbiol. 90, 1–8. 10.1016/S0168-1605(03)00158-2 14672825

[B85] Zhang J. Wang Y. Wang X. Yang K. Yang J. (2003). An improved method for rapidly extracting total RNA from Vitis. J. Fruit Sci. 20, 178–181.

[B86] Zhong S. Joung J.-G. Zheng Y. Chen Y.-R. Liu B. Shao Y.. (2011). High-throughput illumina strand-specific RNA sequencing library preparation. Cold Spring Harb. Protoc. 2011:pdb.prot5652. 10.1101/pdb.prot5652 21807852

[B87] Zhu J. H. Verslues P. E. Zheng X. W. Lee B. Zhan X. Q. Manabe Y. (2005). HOS10 encodes an R2R3-type MYB transcription factor essential for cold acclimation in plants (Retracted article. See vol. 107, pg. 13972, 2010). Proc. Natl. Acad. Sci. U.S.A. 102, 9966–9971. 10.1073/pnas.0503960102 15994234 PMC1175003

