# Supplementary figures and images for: Insights into the Mechanisms Underlying Ultraviolet-C Induced Resveratrol Metabolism in Grapevine (V. amurensis Rupr.) cv. “Tonghua-3”
Source: Front Plant Sci. 2016 Apr 19;7:503. doi: 10.3389/fpls.2016.00503 (PMC4835806; doi:10.3389/fpls.2016.00503)

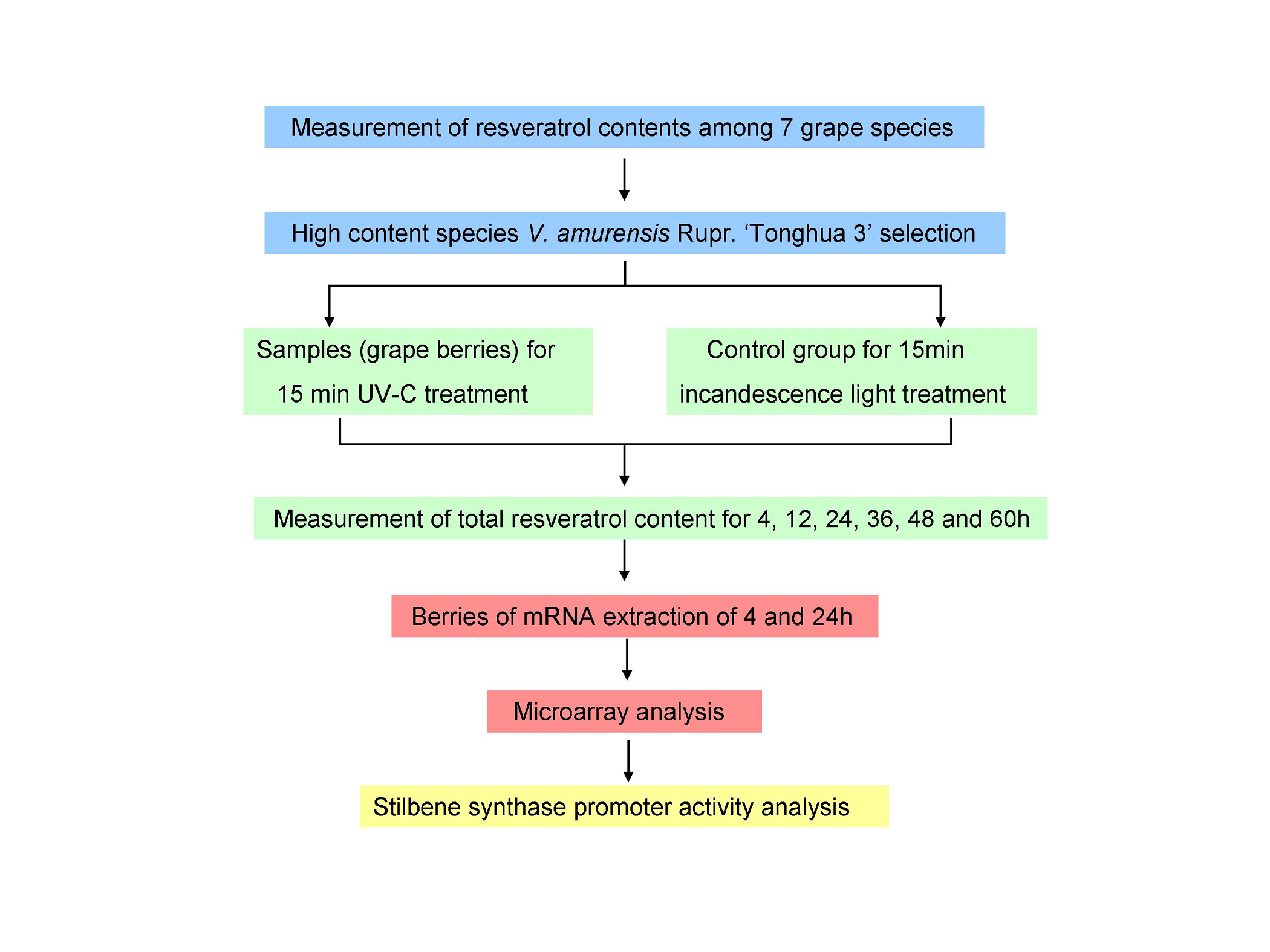

Supplement: Figure S1 — Overview of the experimental design in this study. [file Image1.TIF]

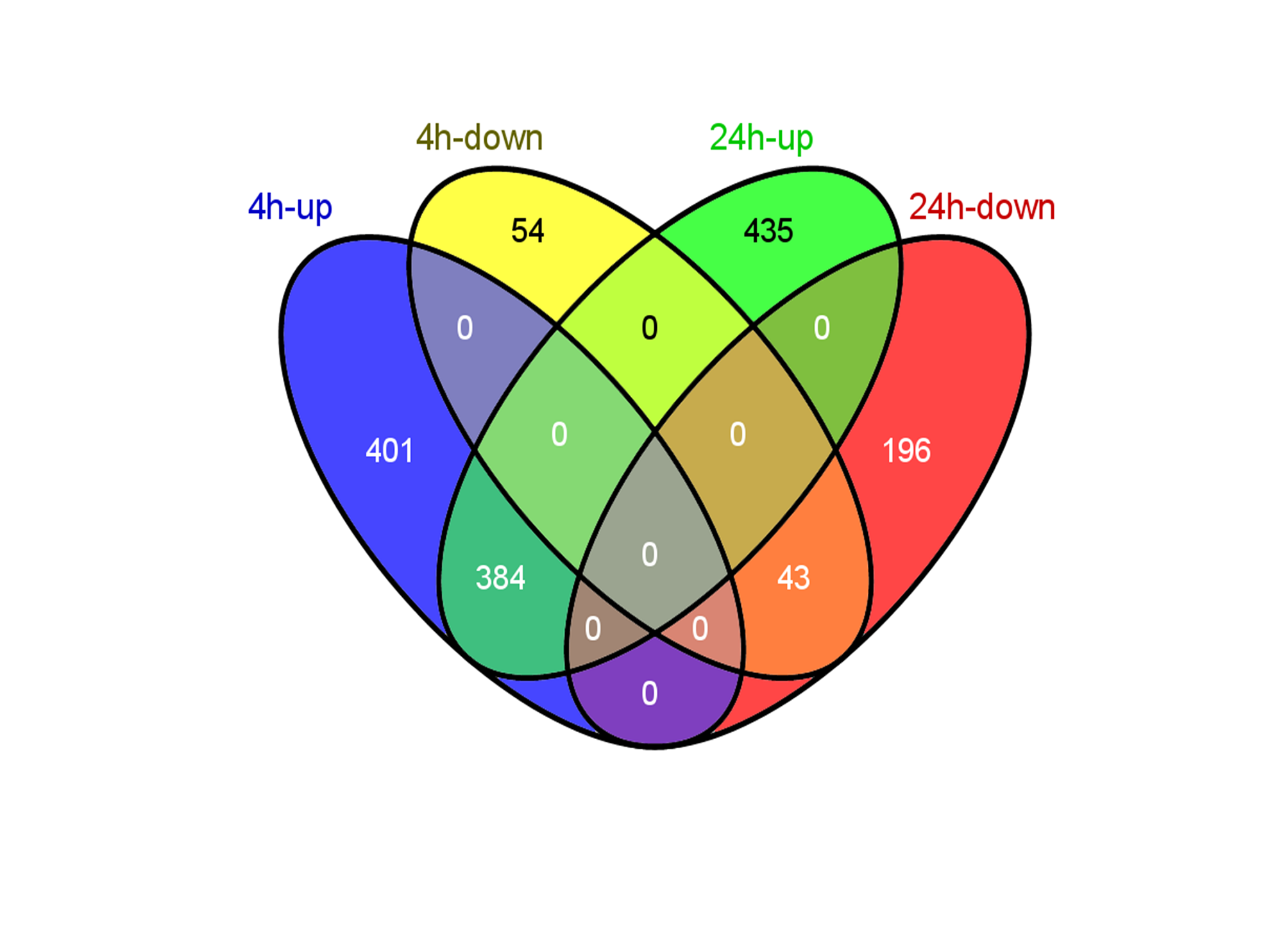

Supplement: Figure S2 — Venn diagrams showing the distribution of RNA-Seq data. [file Image2.TIF]

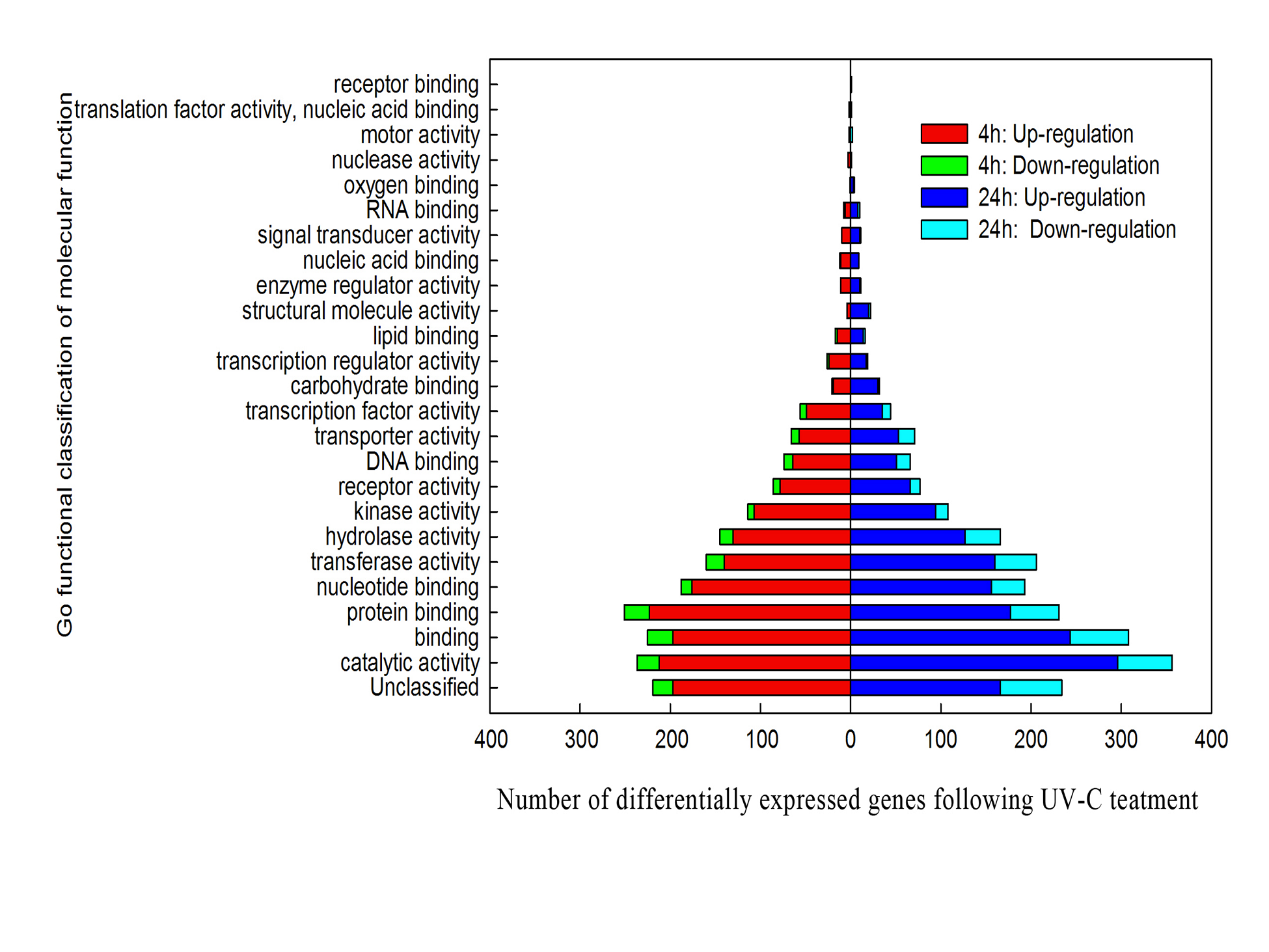

Supplement: Figure S3 — Functional categorization of differentially expressed genes after UV-C treatment within the “molecular function” category of Gene Ontology (GO). [file Image3.TIF]

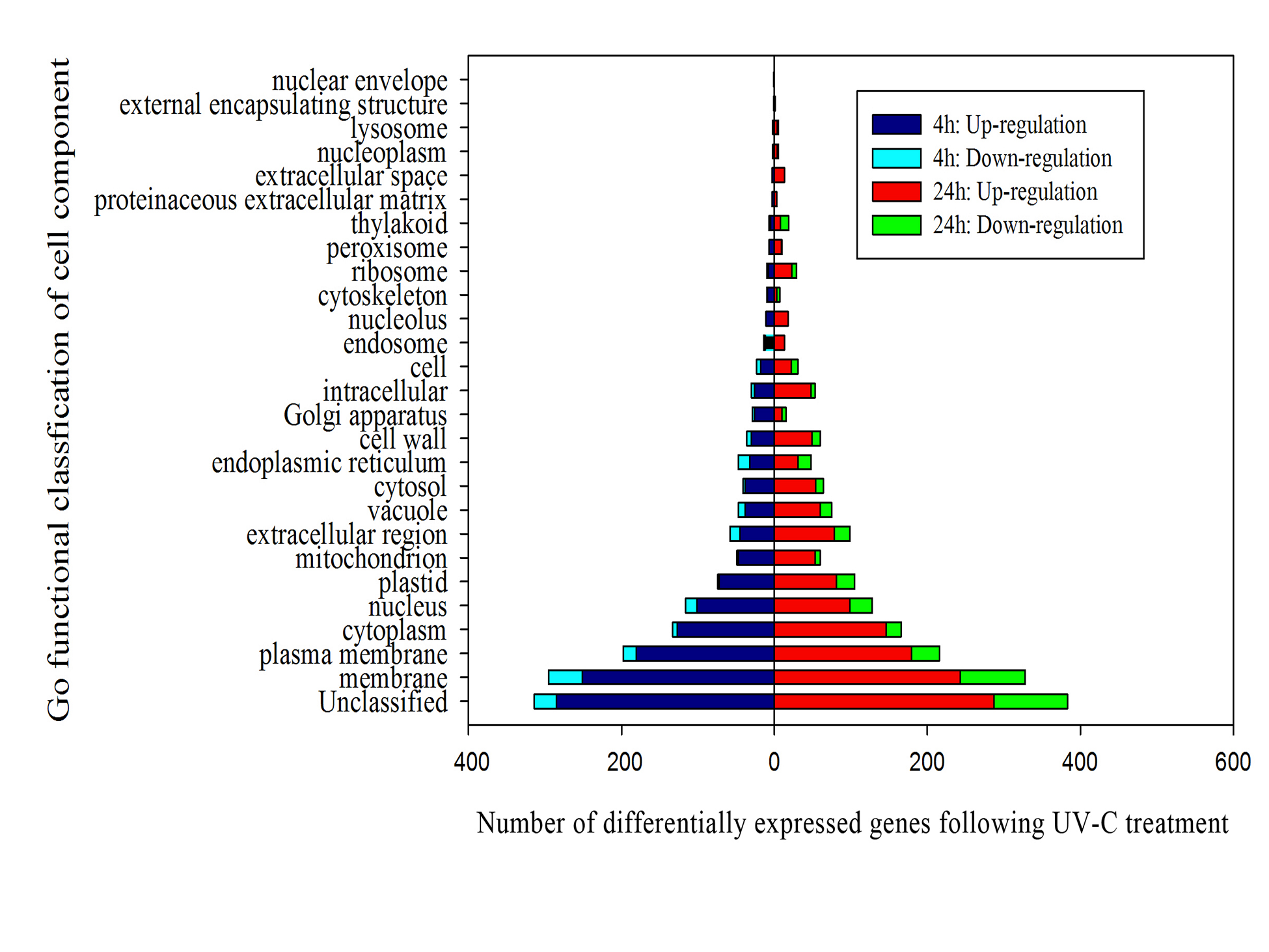

Supplement: Figure S4 — Functional categorization of differentially expressed genes after UV-C treatment within the “cellular component” category of Gene Ontology (GO). [file Image4.TIF]

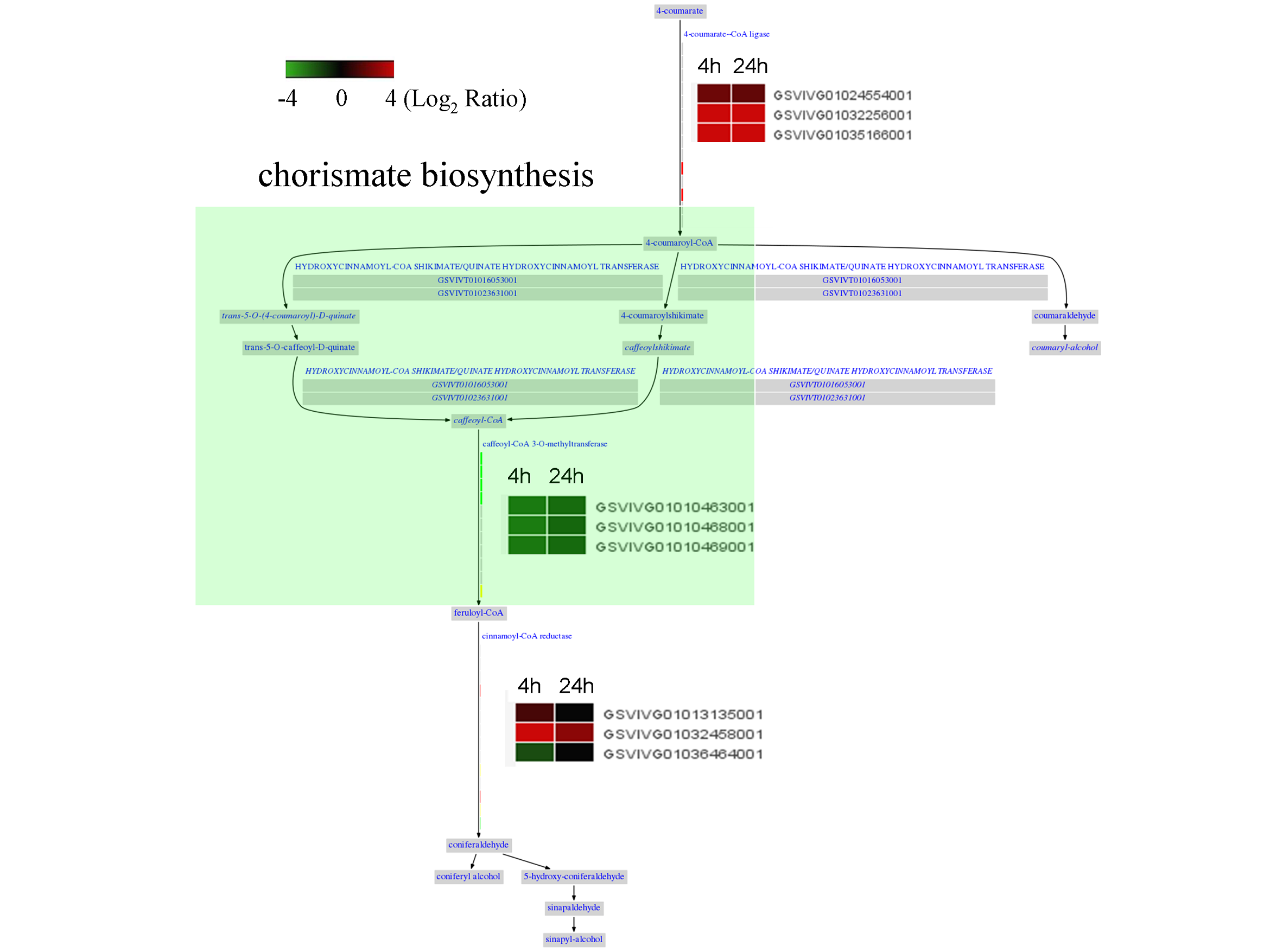

Supplement: Figure S5 — Changes in transcript levels of genes involved in chorismate biosynthesis following UV-C treatment. Different shades of red and green show the extent of the change according to the color bar provided (log2 ratio of control); black indicates no change; gray indicates no transcript detected. [file Image5.TIF]

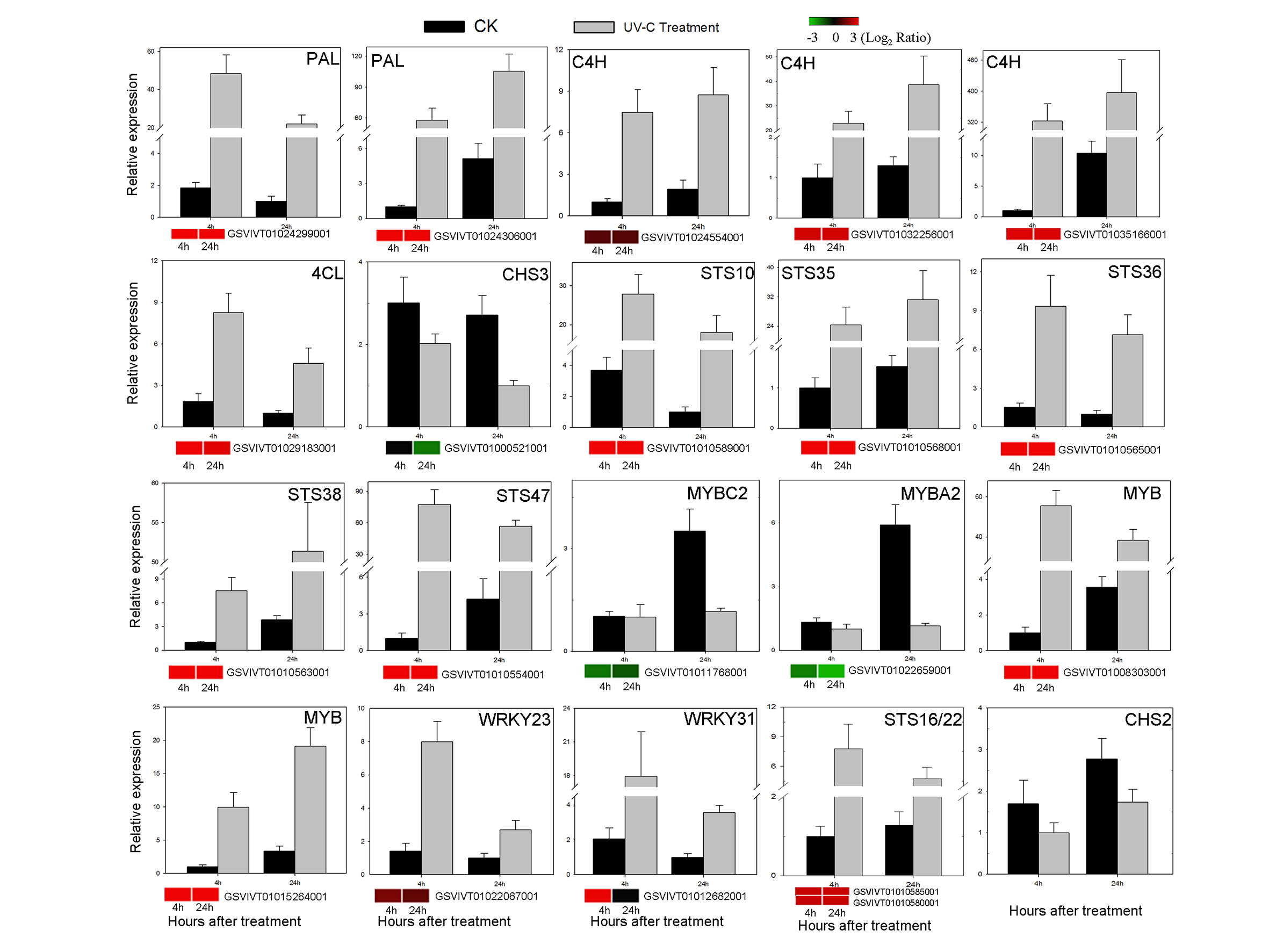

Supplement: Figure S6 — Verification of RNA-Seq results by qRT-PCR analysis of the expression of 20 genes. The grape GAPDH gene (GenBank accession CB973647) was used as the reference gene. Histograms represent abundance assessed by qRT-PCR data, reported as means ± SE of two biological replicates (each biological replicate comprised three technical replicates). Heat maps represent changes in gene expression. The color scale represents relative expression levels, with red denoting up-regulation, green denoting down-regulation and black denoting no change. [file Image6.TIF]

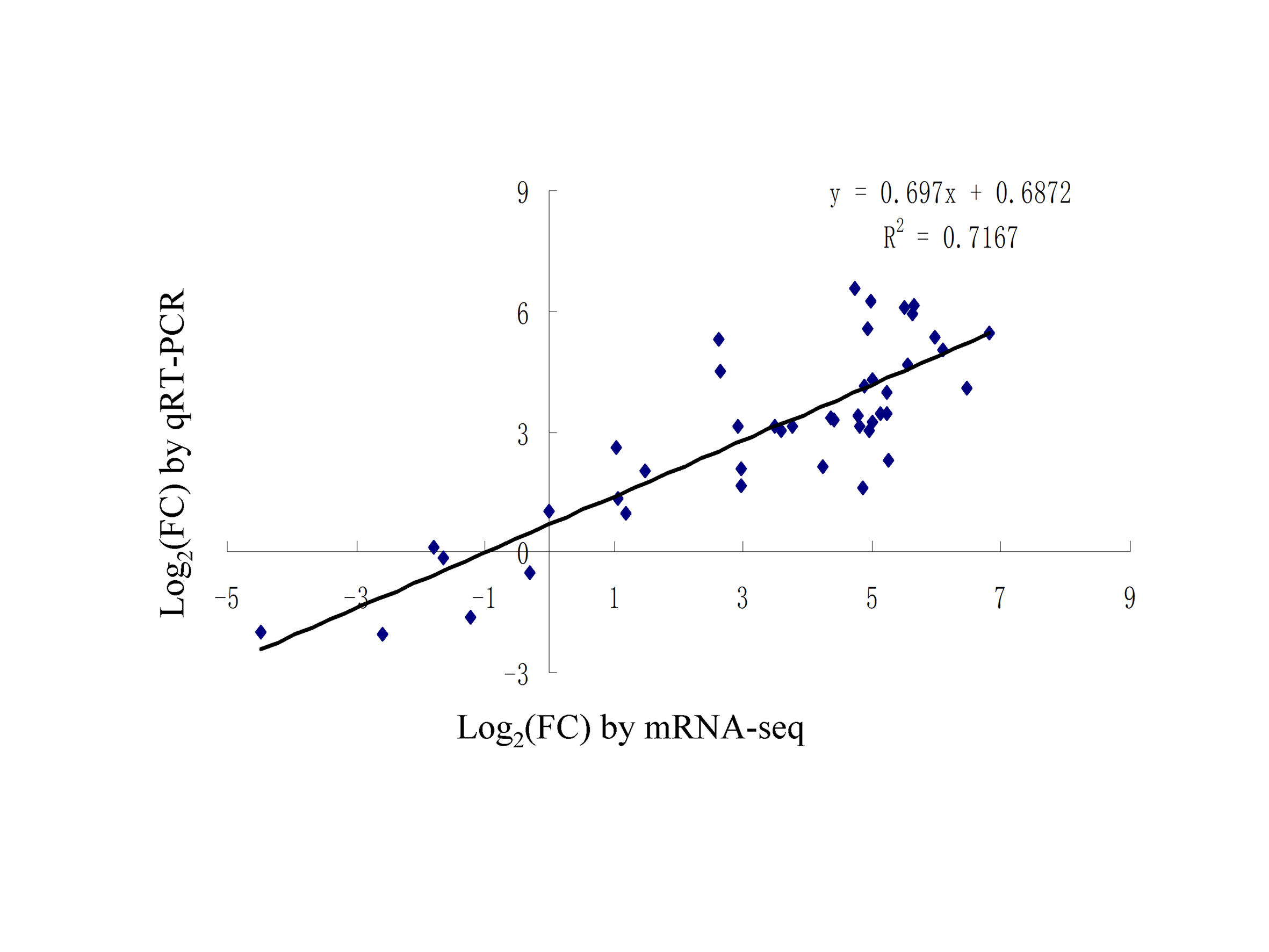

Supplement: Figure S7 — Comparison of the log2 (fold-change; FC) of 20 selected transcripts using RNA-Seq and qRT-PCR analyses. [file Image7.TIF]

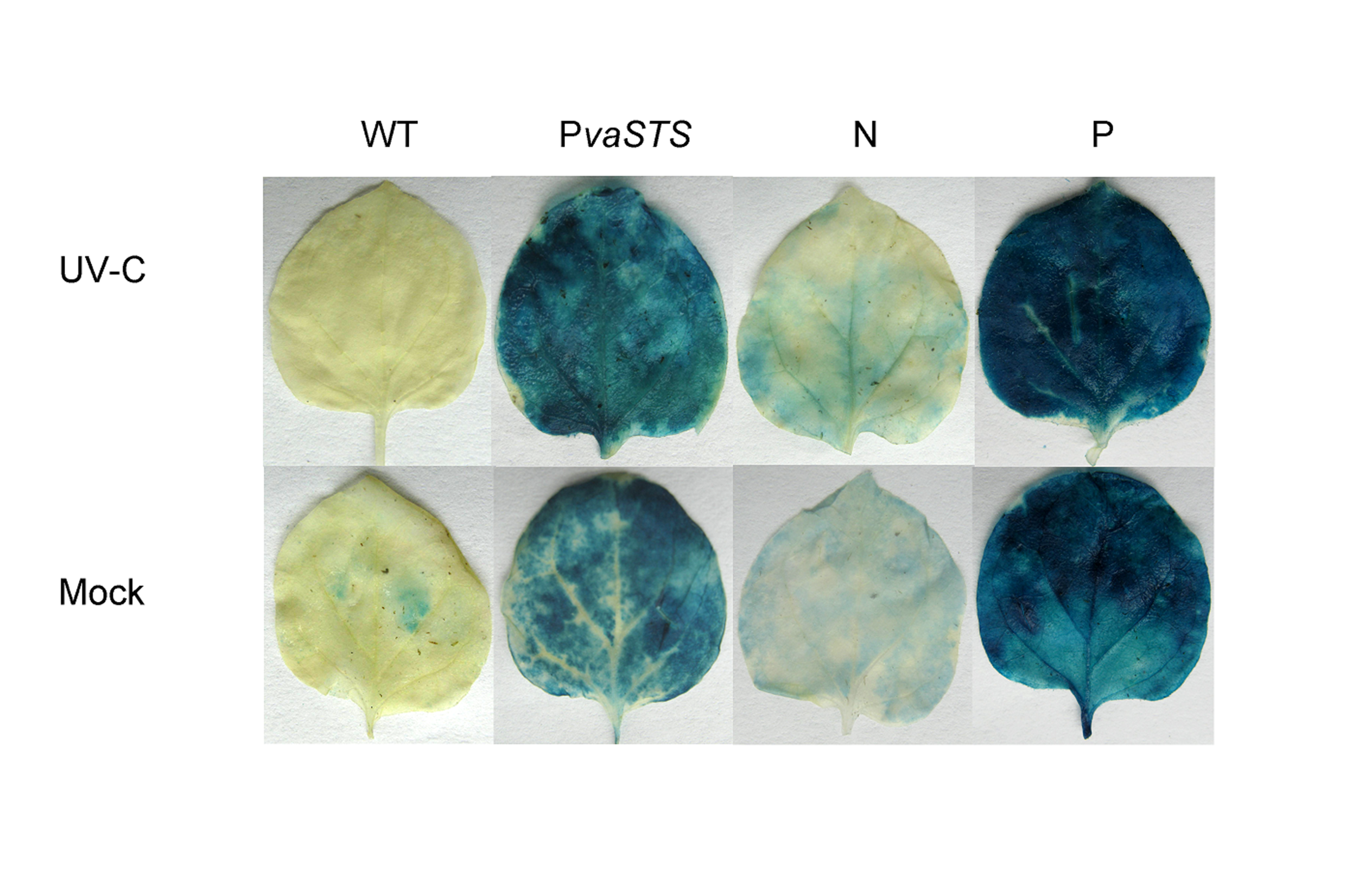

Supplement: Figure S8 — Histochemical assay for GUS expression in transiently transformed leaves of Nicotiana benthamiana after UV-C treatment. Fully expanded leaves from 6 week old N. benthamiana plants were agro-infiltrated with the VaSTS promoter construct at a concentration of OD600 0.6. WT wild type, N represents negative control (pC0380GUS), P represents positive control (pC35SGUS). [file Image8.TIF]

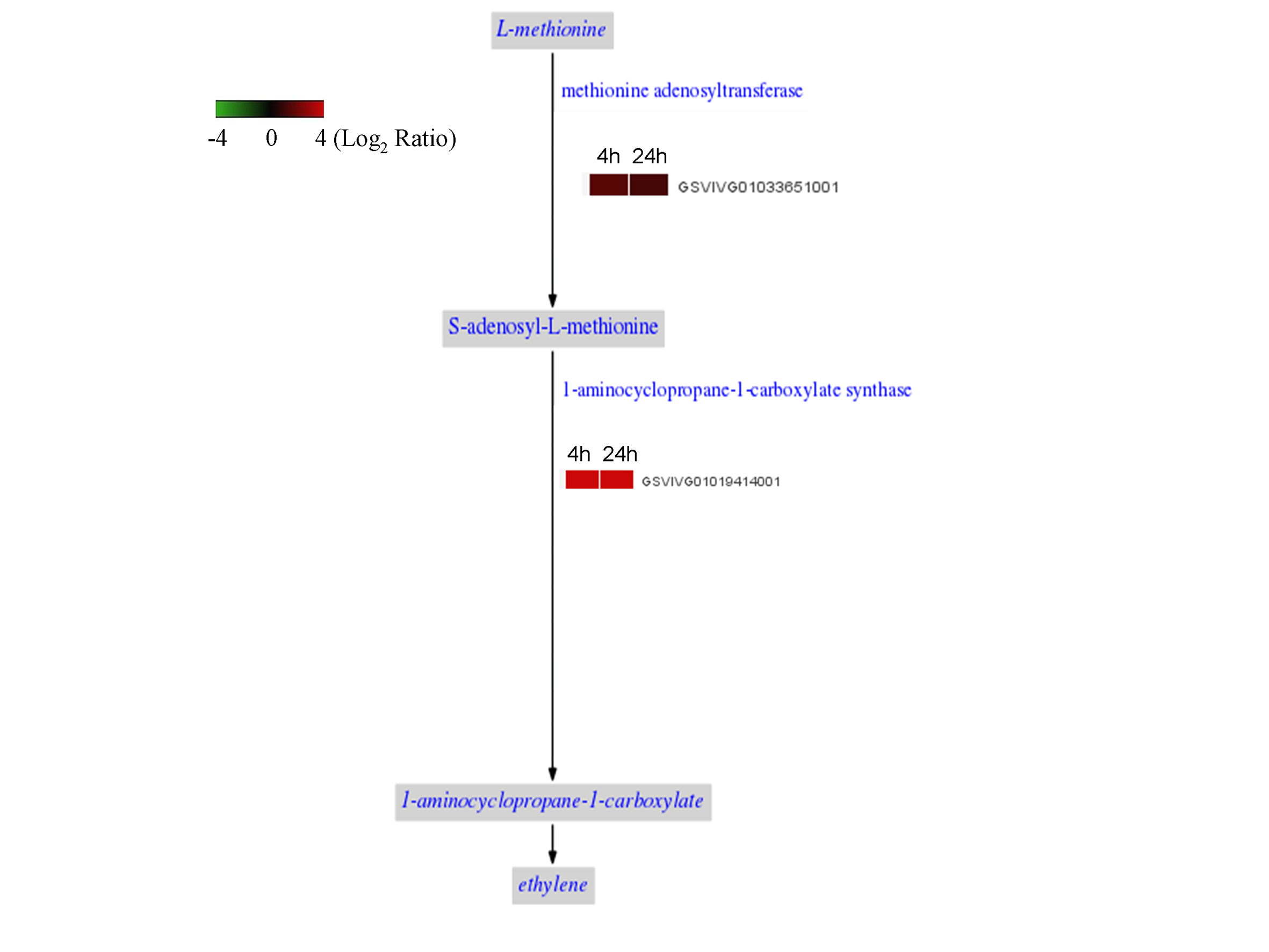

Supplement: Figure S9 — Changes in transcript levels of genes involved in jasmonic acid synthesis following UV-C treatment. Different shades of red and green show the extent of the change according to the color bar provided (log2 ratio of control); black indicates no change; gray indicates no transcript detected. [file Image9.TIF]

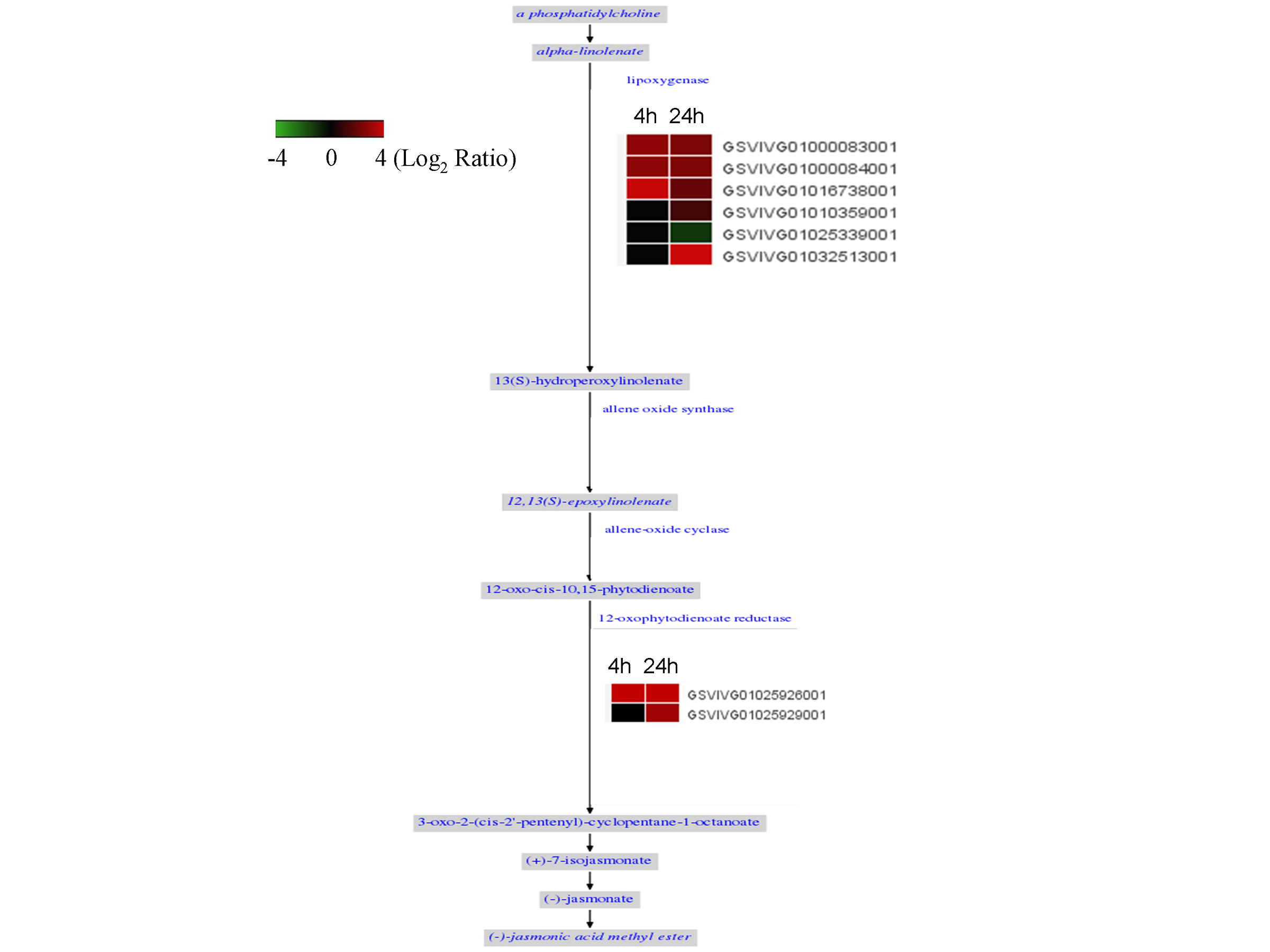

Supplement: Figure S10 — Changes in transcript levels of genes involved in salicylate biosynthesis following UV-C treatment. Different shades of red and green express the extent of the change according to the color bar provided (log2 ratio of control); black indicates no change; gray indicates no transcript detected. [file Image10.TIF]

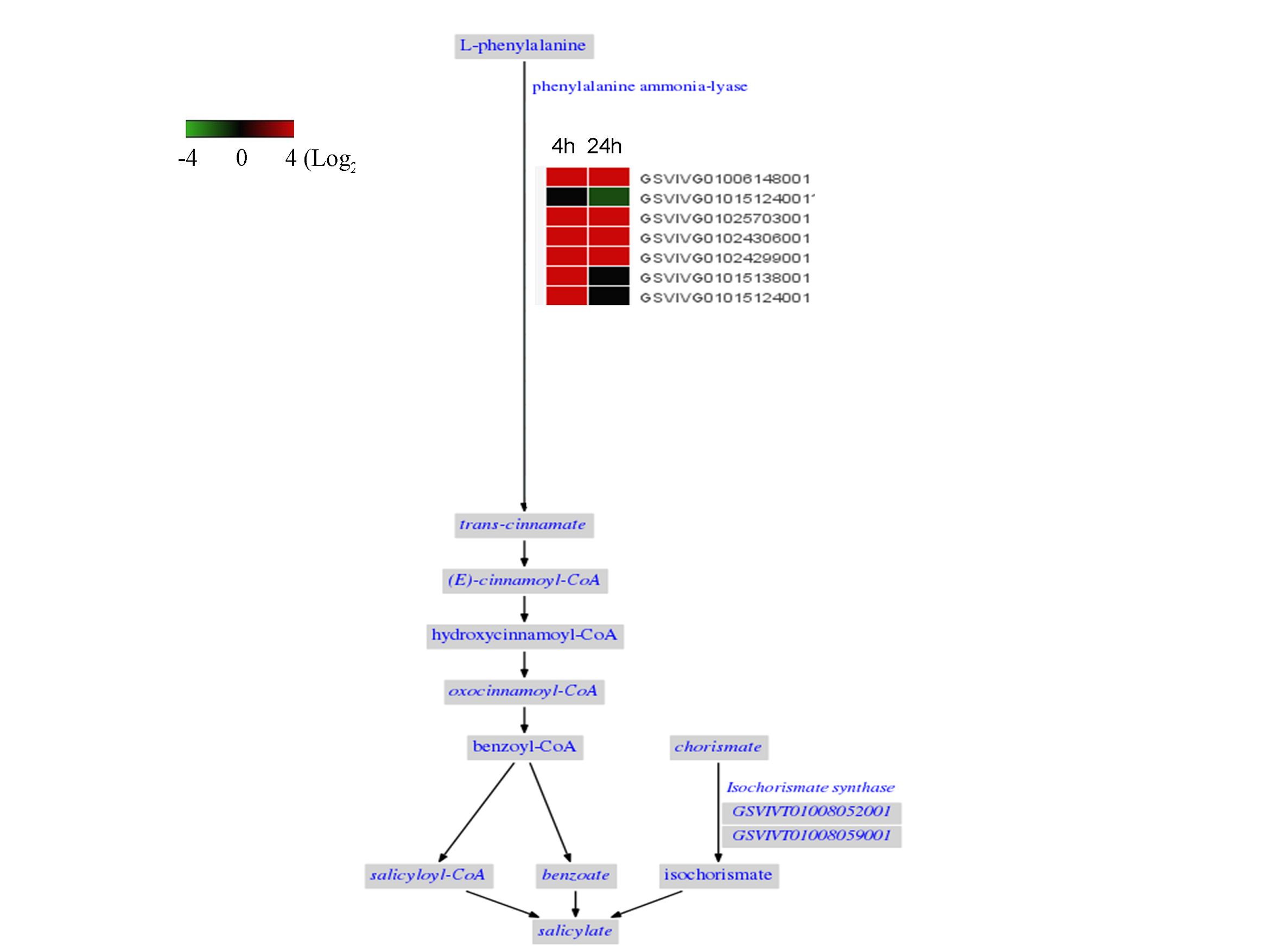

Supplement: Figure S11 — Changes in transcript levels of genes involved in ethylene biosynthesis from methionine following UV-C treatment. Different shades of red and green show the extent of the change according to the color bar provided (log2 ratio of control); black indicates no change; gray indicates no transcript detected. [file Image11.TIF]
